# Development of a Personalized Tumor Neoantigen Based Vaccine Formulation (FRAME-001) for Use in a Phase II Trial for the Treatment of Advanced Non-Small Cell Lung Cancer

**DOI:** 10.3390/pharmaceutics14071515

**Published:** 2022-07-21

**Authors:** Linette T. Oosting, Katka Franke, Michael V. Martin, Wigard P. Kloosterman, Jennifer A. Jamieson, Laura A. Glenn, Miranda W. de Jager, Jacoba van Zanten, Derk P. Allersma, Bahez Gareb

**Affiliations:** 1 Department of Clinical Pharmacy and Pharmacology, University Medical Center Groningen, University of Groningen, Hanzeplein 1, 9713 GZ Groningen, The Netherlands; l.t.oosting@umcg.nl (L.T.O.); j.van.zanten@umcg.nl (J.v.Z.); d_allersma@hotmail.com (D.P.A.); 2 CureVac Netherlands B.V., Matrix Building VII, Science Park 106, 1098 XG Amsterdam, The Netherlands; katka.franke@curevac.com (K.F.); michael.martin@curevac.com (M.V.M.); wigard.kloosterman@curevac.com (W.P.K.); 3 Almac Sciences Scotland Ltd., The Fleming Building, Edinburgh Technopole, Milton Bridge, Penicuik EH26 0BE, UK; jennifer.jamieson@almacgroup.com (J.A.J.); laura.glenn@almacgroup.com (L.A.G.); 4 3D-PharmXchange, Maidstone 48a, 5026 SK Tilburg, The Netherlands; miranda-ext@3d-pxc.com

**Keywords:** formulation, vaccine, neoantigen, peptide, Montanide ISA 51 VG, framome, immunotherapy, non-small cell lung cancer, personalized

## Abstract

Stage III–IV non-small cell lung cancer (NSCLC) is a devastating disease characterized by a poor prognosis. NSCLC tumors carry genetic mutations, which can lead to the expression of altered protein sequences. Peptides originating from mutated proteins and bound to MHC molecules on the tumor cell surface are referred to as neoantigens, as they are tumor-specific and not expressed in normal cells. Due to their tumor specificity, neoantigens have a strong potential to induce an anti-tumor immune response and have been investigated for development of personalized therapeutic cancer vaccines. The current study describes the development of a clinical grade neoantigen vaccine formulation (FRAME-001) intended as immunotherapy in advanced NSCLC in combination with the immune checkpoint inhibitor pembrolizumab. The detection of aberrant tumor-specific transcripts as well as an algorithm to select immunogenic neoantigen peptides are described. Subsequently, selected neoantigen peptides were synthesized with a high throughput synthesis platform and aseptically formulated under good manufacturing practice (GMP) conditions into four aqueous peptides mixtures that each contained six neoantigen peptides. A validated stability-indicating analytical method was developed in which we considered the personalized nature of the formulation. An extensive stability study performed either at −25 °C or −80 °C showed that the formulation was stable for up to 32 weeks. The formulation was mixed with the vaccine adjuvant Montanide ISA 51 VG, which yielded the final vaccine emulsion. The stability of the vaccine emulsion was demonstrated using microscopic examination, differential light scattering, and the water-drop test. The presented data show that FRAME-001 is a feasible personalized vaccine formulation for the treatment of stage III–IV NSCLC. The presented data may give guidance in the development of novel personalized therapeutic vaccines since this formulation strategy could be used for any cancer indication.

## 1. Introduction

Non-small cell lung cancer (NSCLC) accounts for 80% of all lung cancer cases and is in about 55% of cases diagnosed at an advanced stage [1,2]. NSCLC tumors accumulate a large number of somatic mutations during their development, mainly related to tobacco smoking [3,4]. The recent advancements in the treatment of NSCLC are due to the improved understanding of the underlying mechanisms of the disease, including genomics and interactions between cancer cells and the immune system. The major breakthroughs in the treatment of some cancer types, including NSCLC, have been achieved by introducing immune checkpoint inhibitors (ICI). By blocking the inhibitory effect of the immune checkpoint, the anti-tumor response of tumor-reactive T cells is maintained. Presence of infiltrating lymphocytes such as CD8+ cytotoxic T cells and CD4+ T helper cells in the tumor microenvironment and their further expansion are important factors contributing to the clinical response of ICI in NSCLC [5]. However, despite encouraging results of ICI treatment either in monotherapy or combined with chemotherapy in NSCLC, only the minority (20–30%) of patients derive durable clinical benefit [6].

The mutations that a tumor accumulates in its genome can lead to the formation of tumor-specific antigens, referred to as neoantigens. Neoantigens differ in their amino acid sequence from proteins expressed in healthy cells and can therefore give rise to highly immunogenic epitopes (cross-)presented through major histocompatibility complexes (MHC) on tumor cells and on professional antigen presenting cells. These epitopes can be recognized by T cells as foreign, evoking a powerful anti-tumor T cell responses [7]. The administration of such epitopes in the form of a therapeutic cancer vaccine with neoantigen-based synthetic long peptides has been demonstrated to be feasible and showed first signs of efficacy in several (pre)clinical studies [8,9,10,11,12,13]. The ability of therapeutic cancer vaccines to induce antigen-specific T cell immune response against the tumor and the ability of ICIs to increase anti-tumor activity of CD8+ T cells led to the combination of these two therapeutic strategies [12,14,15,16,17].

We have developed a novel method to identify and select neoantigens from patient tumor material. The method applies a novel bioinformatics pipeline that is combined with whole genome sequencing (WGS) and short- and long-read RNA sequencing (Martin et al., manuscript in preparation). This method allows for the selection of tumor-specific neoantigens derived from neo-open reading frames, which are encoded in the tumor genome as a result of a variety of genomic alterations including small insertions/deletions and larger structural variations. Such genomic alterations lead to the translation of novel open reading frame peptides (NOPs), which are then the source of neoantigen sequences containing a number of potentially immunogenic epitopes. 

We have identified that about 90% of lung cancer patients carry at least one of the above-described neoantigens (NOPs) with a number of potential immunogenic epitopes in their tumors. The administration of a personalized vaccine based on multiple selected peptides tailored to the individual patient has the potential to induce de novo immune responses. In addition, it may stimulate the pre-existing anti-tumor immune responses, which together can lead to an increased infiltration of cytotoxic T cell in the tumor microenvironment and draining lymph nodes. To further increase the therapeutic potential, the vaccine may be administered together with an adjuvant and an ICI such as pembrolizumab to enhance the immune response against tumor neoantigens.

One of the commonly used adjuvants for (cancer) vaccines is Montanide ISA 51 VG (hereafter: Montanide). Montanide is an emulsification agent consisting of a mineral oil-surfactant mixture. For a stable emulsion that is suitable for subcutaneous injection, it should be mixed in a 1:1 ratio with the aqueous phase that contains the antigen. Research shows that a combination of vaccine antigens together formulated with Montanide enhances the innate and adaptive immune response, which thereby increases the therapeutical potential of the vaccine. The enhanced immunological response is thought to be the result of injection site-specific inflammation, antigen depot formation, the recruitment of professional antigen presenting cells, and lymphocyte entrapment [18,19].

There are several major challenges associated with personalized neoantigen-based therapeutic cancer vaccines. First, the selection of the amino acid sequences that would contain the most relevant and immunogenic epitopes based on a tumor biopsy of a given patient has to be performed. Second, the selected peptides need to be synthesized from single amino acid starting materials, which necessitates a high throughput manufacturing and analytical test platform rather than product-specific processes and methods. From a stability point of view, single freeze-dried peptide formulations are desired. However, in view of administering multiple peptides (i.e., 20–24 neoantigens) formulated with the vaccine adjuvant Montanide at once, an aqueous solution containing several peptides in a mixture is desirable and more patient friendly (i.e., fewer injections). Thus, stable aqueous peptide mixtures need to be manufactured from the synthesized peptides. Besides the potential solubility, compatibility, and stability issues associated with aqueous peptide mixtures, a validated stability-indicating analytical method should be in place for the quality control analyses and stability study. The development and validation of the analytical method is challenging since the manufactured peptides differ from batch to batch based on the patient’s tumor biopsy material. Finally, a limited time frame for the entire manufacturing process, which must take place under good manufacturing practice (GMP) conditions, is a prerequisite to minimize the time between patient screening and vaccine administration. 

The objective of the study was to develop a personalized neoantigen-based vaccine formulation (FRAME-001) for use in a phase II trial for the treatment of advanced NSCLC (clinical trial: NCT04998474). FRAME-001 consisted of four peptide mixtures each containing six peptides, which were intended to be emulsified with the vaccine adjuvant Montanide prior to administration. First, single neoantigens were detected in tumor material applying genomic and transcriptome sequencing analyses, and a novel bioinformatics pipeline. Subsequently, the selected neoantigens were synthesized and formulated into freeze-dried peptide mixtures (drug substance) that were suitable for further GMP processing into aqueous peptide mixtures (drug product). A risk-based approach was utilized to develop a GMP manufacturing process and validated stability-indicating analytical method in which we considered the personalized nature of the drug product. The Montanide compatibility with the drug product was investigated with an emulsion stability study. Finally, an extensive stability study was conducted in which the drug product was stored either at −25 °C or −80 °C for a storage period of 32 weeks. 

## 2. Materials and Methods

### 2.1. FRAME-001 Characteristics and Specifications

For each patient, a set of up to 24 patient-specific neoantigen peptides based on tumor biopsy data are synthesized and mixed to form up to four freeze-dried mixtures that each contain up to six peptides (drug substance). The four patient-specific peptide mixtures are then individually formulated into an aqueous buffer (1.40 mL) at a concentration of 143 µg/mL per peptide (drug product). FRAME-001 is the neoantigen vaccine emulsion that is obtained after emulsifying the individual aqueous peptide mixtures with the adjuvant Montanide in a 1:1 ratio (0.90 mL:0.90 mL). From this 1.80 mL of emulsion, 1.40 mL is withdrawn in a syringe and administered subcutaneously, which corresponds to a dose of 100 µg per peptide. The four peptide mixtures yield four vaccine emulsions that will be administered to the patient at four different injection sites (i.e., left and right upper arms and legs) on one given vaccination day.

FRAME-001 is intended to be administered as combination therapy together with the ICI pembrolizumab over a period of 9 weeks. In total, there will be four vaccination days with an interval of 3 weeks between each vaccination day (t = 0, t = 3 weeks, t = 6 weeks, and t = 9 weeks). Thus, a peptide mixture stability of at least 9 weeks is a prerequisite for FRAME-001.

**Figure 1:**
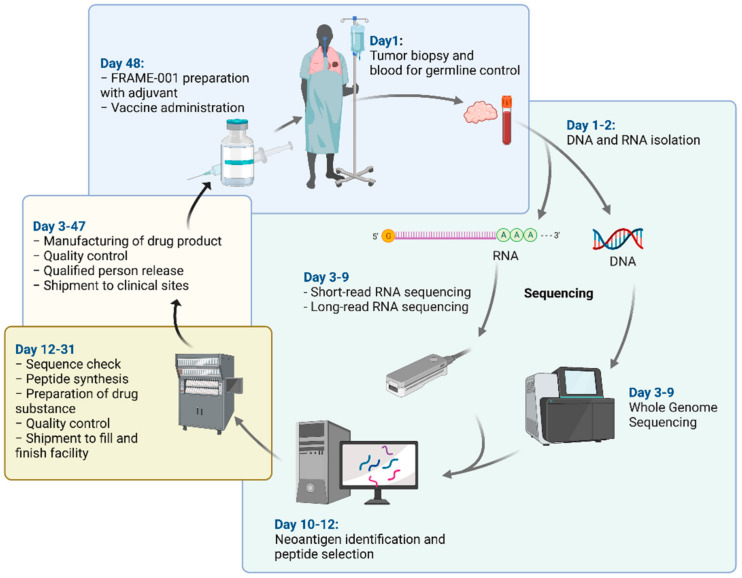
Schematic representation of the entire manufacturing process of FRAME-001.

The timeline for the entire manufacturing process of FRAME-001 is 48 business days, from patient screening until vaccine administration (Figure 1). At day 1, biopsy and blood sample are taken from eligible patients for DNA and RNA analysis. At day 12, the neoantigen peptides are selected and submitted for synthesis. At day 31, the synthesized and quality-controlled personalized freeze-dried peptide mixtures are shipped to the sterile fill and finish facility to further formulate into aqueous peptide mixtures, which are released for clinical use at day 47. At day 48, the aqueous peptide mixtures are emulsified with the vaccine adjuvant Montanide at the local pharmacies of the clinical trial sites and administered subcutaneously to the patient.

The specifications of FRAME-001 are given in Table 1. Since FRAME-001 consists of up to four patient-specific peptide mixtures that each contain up to six individual neoantigens, the present study investigated four peptide mixtures that each contain six individual peptides as the most optimal scenario.

### 2.2. NOP Identification

The method of NOP identification was described in more detail in Martin et al. (manuscript in preparation). Briefly, for each sample paired tumor-normal WGS and tumor long-read (Oxford Nanopore Technologies, Oxford, UK) and short-read (Illumina, San Diego, CA, USA) RNA sequencing was performed. These data were integrated with a novel bioinformatics pipeline named FramePro, which used somatic genetic variations to construct a tumor specific reference genome. The transcriptome data were remapped to this reconstructed genome and the protein sequence of RNA isoforms were predicted using annotated gene start sites. The novel protein coding sequences within these predicted peptides were extracted to form a set of all NOPs, termed the framome, which are specific for each tumor. Expression percentiles for each NOP were generated by multiplying the supporting long read counts by the inverse of the tumor purity, and then through a comparison to the protein coding gene expression levels quantified by FLAIR [20]. MHC binding epitopes were predicted using netMHCpan4.1 [21] using tumor-specific human leukocyte antigen (HLA) alleles predicted based on WGS using Polysolver [22]. A peptide with a netMHCpan4.1 eluted ligand (EL) score less than two was considered a predicted binder.

### 2.3. Peptide Selection

NOPs smaller than 5 amino acids were excluded. Neoantigen peptide sequences originated in NOPs were appended with enough upstream wild-type (WT) sequence to produce a final sequence length of 35 amino acids with all NOPs having at least four WT amino acids prepended. These parent peptides were tiled with 30 amino acid long peptides with a minimum overlap of 10 amino acids. Each of these sub-peptides were given a score based on the following Formula (1):

S = V × E × F (1)

where V is the genomic variant allele frequency (VAF) of the parent peptide, E is the expression percentile of the parent peptide, and F is the WT fraction of the sub-peptide. The 24 peptides with the highest score were selected for inclusion in the vaccine design, manufacturing, and analysis. These peptides were then trimmed of N-terminal glutamine and C-terminal cysteine, histidine, and proline to produce peptides amenable for manufacturing. Additional peptide trimming/extension of one or two amino acids on the N/C termini was carried out based on expert inspection to improve solubility.

### 2.4. Peptide Synthesis 

The single peptides and peptide mixture were synthesized under GMP conditions using a platform manufacturing process, which comprises the following steps: (1) parallel peptide synthesis, (2) peptide cleavage, (3) peptide purification, (4) 0.22 µm filtration and freeze-drying, (5) peptide mixture preparation, and (6) shipping. Supplementary Figure S1 shows a flow chat of the manufacturing platform.

#### 2.4.1. Parallel Peptide Synthesis

**Figure 2:**
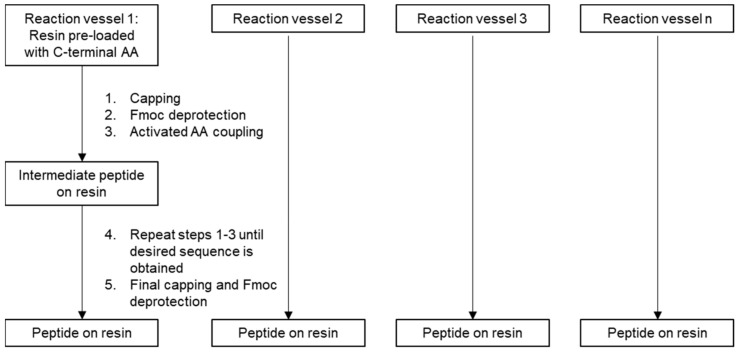
Schematic representation of the automated peptide synthesis process.

Each group of patient-specific peptides were synthesized concurrently on a multi-channel parallel peptide synthesizer using a Symphony X (Gyros, Tucson, AZ, USA) by solid phase peptide synthesis (SPPS) using the Fmoc (9-fluorenylmethyloxycarbonyl) protection strategy (Figure 2). Prior to initiating synthesis, the patient-specific peptide sequences were assessed for the presence of any structural moieties which may have a negative impact on manufacturability or intrinsic stability. Where these moieties were present, the peptide sequence were adjusted to the minimum extent possible to remove the moiety of concern whilst still fully preserving the patient-specific characteristics of the sequence. 

Once the sequence assessment was concluded, resin pre-loaded with the C-terminal amino acid specific to each peptide was placed in a reaction vessel on the parallel synthesizer. A Wang polystyrene resin (100–200 mesh) was employed which comprises a copolymer (styrene—1% divinylbenzene) solid support with a 4-hydroxybenzyl alcohol linker. The amino acid residues were incorporated by a series of capping (acetic anhydride in dimethylformamide (DMF)), Fmoc deprotection (piperidine in DMF), and amino acid coupling cycles (amino acid, Oxyma Pure (ethyl 2-cyano-2-(hydroxyimino) acetate) and diisopropylcarbodiimide (DIC) in DMF). Oxyma Pure and DIC were used as the coupling reagents for activation of the amino acid C-terminus. An amino acid:Oxyma Pure mixture was transferred to the reaction vessel followed by DIC solution and the combined solution was mixed (by nitrogen bubbling of the solution) to form the amino acid activated ester which reacted with the resin bound amino terminus of the growing peptide. The peptide chain was assembled on the resin by repeating the coupling process until all of the amino acids required for the sequence were incorporated. 

Excess amino acid and all reagents were removed by DMF washing at each sequential assembly step. When assembly of the desired sequence was complete, the resin was subjected to a final capping and Fmoc deprotection cycle, followed by a wash with dichloromethane.

#### 2.4.2. Peptide Cleavage and Deprotection

After on-resin assembly of the peptide sequences within the synthesizer multiple channels, each peptide was cleaved from its resin with concomitant side chain deprotection using a cleavage cocktail comprising trifluoroacetic acid (TFA) and scavenger reagents. The scavenger reagents were triisopropylsilane (TIS)/water/dimethylsulfide (DMS)/1,2-ethanedithiol (EDT). This provided the crude peptide directly as a C-terminal carboxylic acid. The cleavage mixture was filtered to remove the spent resin, and the filtrate was mixed with chilled diethyl ether to precipitate the crude peptide. The crude peptide was isolated from the diethyl ether by centrifugation. The isolated pellet was washed repeatedly with diethyl ether to remove cleavage reagents, byproducts, and scavengers, and was subsequently dried under vacuum and analyzed for area % purity and identity (in-process control (IPC) 1, Supplementary Figure S1).

Purity and identity were analyzed with ultra-performance liquid chromatography (UPLC) coupled to a mass spectrometer (MS). The method consisted of a C18 media column with an acetonitrile/water solvent system containing TFA as a modifier, and masses were scanned from 50–2000 *m*/*z*.

#### 2.4.3. Peptide Purification

Purification was performed by preparative reverse phase high performance liquid chromatography (HPLC) using a C18 media column with an acetonitrile/water solvent system containing TFA as a modifier. The apparatus, column, flow rate, mobile phase A, and mobile phase B were: Äkta Pure purification system (Cytiva, Marlborough, MA, USA); Phenomenex C18 media column (Phenomenex, Torrance, CA, USA); 20 mL/min; 0.1% TFA in water; 0.1% TFA in acetonitrile, respectively. The purification gradient ran from low to high organic content; the specific gradient applied was dependent upon the hydrophobicity of the peptide being purified. The chromatography fractions were collected, analyzed for area % purity by UPLC (IPC 2, Supplementary Figure S1), and fractions were pooled to achieve a final pool purity of ≥90 area % by UPLC-UV. Purity analysis was performed on an Acquity UPLC H-class (Waters Corporation, Milford, MA, USA) using a suitable C18 column and acetonitrile/water gradient containing 0.1% TFA as modifier. The purity UPLC-UV release test method was qualified prior to use by conducting a phase-appropriate qualification using three reference peptides. The method was qualified for linearity, sensitivity (LOD/LOQ), precision, and sample stability.

#### 2.4.4. Filtration and Freeze-Drying

The pooled chromatography fractions were passed through a 0.22 µm disc filter (Millex, Merck Millipore, Burlington, MA, USA) into a round bottom flask. The flask was attached to a benchtop freeze-drier (Christ, Osterode am Harz, Germany) and dried for a minimum of 12 h at ≤−50 °C at ≤0.2 mbar. The freeze-dried peptides were discharged and analyzed (Intermediate Testing, Supplementary Figure S1) for appearance, area % purity, and identity by UPLC-MS with the analytical method described above.

#### 2.4.5. Peptide Mixture Preparation and Freeze-Drying

The freeze-dried peptides were combined into four peptide mixtures, each containing six peptides. Peptide mixing was carried out on an equi-net weight basis by weighing individual solid peptides, correcting the gross weight for theoretical peptide content (and purity in cases where the peptide purity was <97 area %), and mixing the peptides on a dry basis. Each solid peptide mixture was dissolved/suspended in acetonitrile/water (50:50 *v/v*) to achieve homogenization and the solution/suspension was transferred to a round bottom flask. The four flasks containing the four mixtures were attached to a benchtop freeze drier and dried for a minimum of 12 h as described above. The four freeze-dried mixtures were discharged and analyzed (Release Testing, Supplementary Figure S1) for appearance, area % purity by UPLC-UV, identity by UPLC-MS, peak ratio by UPLC-MS, TFA by HPLC-UV (USP 503.1), bacterial endotoxins (USP 43 <85>), and bioburden (USP 43 <61>).

The four mixtures were packaged into glass type I vials (Ph. Eur. 3.2.1) assembled with butyl stoppers (Ph. Eur. 3.2.9) and aluminum seals. The vials were stored and shipped at −20 ± 5 °C for further processing. 

### 2.5. Development and Evaluation of the Drug Product Production Process

A multidisciplinary prospective risk assessment for the production process of FRAME-001 under GMP condition was performed. Different possible production methods were assessed, i.e., gravimetric, volumetric, single- or multi-syringe, and single- or multi-pipette processes. The potential risks associated with the production method were identified, investigated, and mitigated.

The production process consisted of dissolving the freeze-dried peptide mixtures in the formulation buffer by transferring the solution directly into the vial. This resulted in a peptide concentration of 143 μg/mL per peptide. The peptide mixture solution was then sterilized by a sterile filtration step with a 0.22 μm filter (Millex GV, Merck Millipore, Burlington, MA, USA, reference: SLGV033RS). The sterilized peptide mixture was filled into 6R vials (type I clear borosilicate glass Ph. Eur. 3.2.1) and capped with a bromobutyl stopper (Ph. Eur. 3.2.9) and aluminum seal. The entire production process took place in a laminar air flow cabinet (EU GMP grade A air) situated in an EU GMP grade B background environment. The four representative aqueous peptide mixture batches (FRM001-M1–FRM001-M4) were produced with this production process (yield: 32–34 vials). These four peptide mixture batches were also used for the long-term and in-use stability study (see Section 2.8).

The risks associated with the production process were the volumetric accuracy of the syringe that was used for transferring the formulation buffer into the vial that contained the freeze-dried peptide mixture, potential peptide adsorption to the sterilization filter, the sterility of the formulation, the solubility and stability of the peptides in the formulation buffer, and the compatibility of the peptide mixture with Montanide for a stable emulsion that is suitable for subcutaneous injection. 

Furthermore, the development of a validated stability-indicating analytical method was challenging since FRAME-001 is a patient-specific and personalized formulation that differs from batch to batch (Section 2.6). An additional challenge associated with the analytical method was the absence of an internal standard, which made it impossible to determine the assay value of the manufactured peptide mixtures. These possible risks and challenges were investigated to warrant a robust and qualified production process that complied with EU GMP requirements.

A 60-mL BD Plastipak luer-lock syringe (Becton Dickson, Madrid, Spain, reference: 300865) was used to transfer the formulation buffer into the vial that contained the freeze-dried peptide mixture. The accuracy of the syringe was investigated by withdrawing 49.0 mL of water for injection (WFI) at room temperature according to the measure lines of the syringe. The expelled WFI was then gravimetrically analyzed (*n* = 10). A density of 1.0 g/mL was used for the calculation.

Sterile phosphate-buffered saline (PBS) 10 mM pH 7.0 (Apotheek A15, Gorinchem, The Netherlands, reference: EP00473) was selected as the formulation buffer since it has known acceptability with regard to subcutaneous administration. Furthermore, the solution was buffered and had a neutral pH, which both were expected to be beneficial for the solubility and stability of the peptide. It was also expected to mix well and form a stable emulsion with Montanide since it is an aqueous solution that did not contain any excipients that might interfere with the formation of a stable emulsion.

To investigate whether the peptides adsorbed to the filter that was used for the sterile filtration step, test samples of the peptide mixture solution were taken before and after the filtration step. The peptide identity, peptide peak ratio, peptide purity, potential impurities, peptide retention times, and peptide mixture chromatograms were analyzed with the validated stability-indicating analytical method described in Section 2.6.

Aseptic process qualification was performed by simulating the entire production process with three consecutive media fills. The medium consisted of a sterile tryptic soy broth (Biotrading, Mijdrecht, The Netherlands). After filling, the containers were incubated for 7 days at 22.5 ± 2.5 °C and immediately after for 7 days at 32.5 ± 2.5 °C. A growth promotion tests (Ph. Eur. 2.6.1 and 2.6.12) was conducted after the incubation period of 14 days (Eurofins Bactimm, Nijmegen, The Netherlands). The acceptance criteria were no microbial growth during the 14-day incubation period and a positive growth promotion test according to the designated Ph. Eur. methods after the incubation period completed. 

### 2.6. Validation of the Stability-Indication Analytical Method

A UPLC-MS system was used for the development and validation of the stability-indicating analytical method. The apparatus, column, flow rate, mobile phase A, mobile phase B, mass acquisition, and sample run time were: UPLC UltiMate 3000 RSLC System (Thermo Fisher Scientific, Waltham, MA, USA) coupled to a Q Exactive Orbitrap Mass Spectrometers (Thermo Fisher Scientific, Waltham, MA, USA); Acquity UPLC Peptide CSH C18, 130 Å, 1.7 µm, 2.1 mm × 150 mm (Waters Corporation, Milford, MA, USA); 0.40 mL/min; 0.013% ammonium formate and 0.1% formic acid dissolved in ultrapure water (pH 2.8); 0.013% ammonium formate and 0.1% formic acid dissolved in a mixture of 0.495:0.495:0.10 methanol, acetonitrile, and ultrapure water; masses scanned from 300.0–1900.0 *m*/*z*; 15 min; respectively. The mobile phase gradient started at t0 min with 98% of mobile phase A that was gradually adjusted to 40% at t9 min. At t9.1 min, mobile phase A was set at 2% till t11.1 min. At t11.2 min, mobile phase A was set at 98% until the end of the sample run time.

The peptide sequence, theoretical molecular weight, and peptide concentration of the reference standard were APYTFGQGT-K*-[K* = K(13C,15N)], 100 µg/mL in PBS, and 1077.17 amu, respectively (SIL Adalimumab, Grenoble, France, reference: SB042, lot: 8037_A). The reference sample was analyzed in triplicate on three consecutive days for the system suitability test.

Three representative peptides were selected for the validation analytical method of the drug product. The peptides were selected based on the experience and knowledge from the drug substance high throughput analytical test platform. The peptides were chosen to cover a range of chain lengths, isoelectric point (pI) values, and hydrophobic/hydrophilic properties. The peptide sequence, theoretical molecular weight, and pI values of the three individual peptides were FRM001P02: SVTNTSLAHELWKVPLHLKTSLLRPAHLRD, 3432.9 amu, pI = 10.6; FRM001P11: SAMEKRGARETQQQRKRVFRGRGVNSAK, 3231.7 amu, pI = 12.5; FRM001P23: VTLEERLDKACEPGVDYDGQTGVIHFQPRG, 3329.6 amu, pI = 4.4 (Supplementary Table S1). 

For each peptide, six stock concentrations in the range of 5–225 µg/mL dissolved in PBS were made. The linear relationship of the calibration curves was investigated by regression analysis. The linear correlation coefficient (R^2^), goodness of fit, and lack of fit were analyzed. For the accuracy and precision tests, samples of 50 µg/mL, 150 µg/mL, and 225 µg/mL were analyzed in triplicate on three consecutive days to determine the closeness of agreement of the measurements compared to the analyzed concentrations (accuracy % bias) and repeated experiments (relative standard deviations (RSD)). For the sensitivity test, peptide samples at 5 µg/mL were analyzed in triplicate on three consecutive days and the signal-to-noise (S/N) ratio was calculated from representative chromatograms. For the freeze–thaw experiment, samples at 150 µg/mL for each of the three peptides were prepared and analyzed in fivefold on three consecutive days after each sample was subjected to two freeze–thaw cycles before the analysis was initiated.

The peptide mixtures (FRM001-M1–FRM001-M4) that were manufactured under GMP conditions were used for the specificity tests. The four aqueous peptide mixtures each contained six different peptides. The masses and pI of the 20 unique peptides ranged from 2967.5–3625.9 amu and 4.2–12.7, respectively. Each peptide had a sequence of 28–31 amino acid. Supplementary Table S1 gives the characteristics of the 20 different neoantigen peptides. 

### 2.7. Vaccine Emulsion Formulation and Stability

The formulation buffer as well as the aqueous peptide mixtures manufactured under GMP conditions were both separately emulsified with Montanide with the two-syringe and i-connector (Prompela, Albert II, Monaco, reference: ODG0015ST) method described by the supplier (Seppic S.A., La Garenne-Colombes, France). The emulsification process consisted of a pre-emulsification step at low speed (20 slow cycles) and the emulsification step at high-speed (40 fast cycles) in two 2-mL syringes (B. Braun Injekt, Melsungen, Germany, reference: 4606701V).

For the pre-emulsification step, 0.90 mL of formulation buffer was withdrawn in syringe 1 and 0.90 mL of Montanide was withdrawn in syringe 2. Both syringes were connected to the i-connector and the content of syringe 1 was transferred via the i-connector to syringe 2. The entire content of syringe 2 was then transferred via the i-connector to syringe 1. One cycle was defined as the transfer of the entire content from one syringe to the other through the i-connector, and back again to the first syringe. The first 20 slow cycles were performed with a slow rhythm of 4 s to transfer the content from one side to the other. Thus, a complete cycle required 8 s. 

The following emulsification step consisted of 40 cycles at high speed. The transfer speed was increased to 1 s. Thus, a complete cycle required 2 s. The formed emulsion had a white, vicious, creamy appearance (Supplementary Figure S2). The entire emulsion was then transferred to a single syringe and disconnected from the i-connector. 

For the emulsion stability study, a 25G 5/8 inch sterile subcutaneous injection needle (Beckton Dickson Microlance 3, Vianen, The Netherlands, reference: 300600) was connected to the syringe to investigate whether the emulsion remained stable after the emulsification procedures and the following expulsion of the emulsion through the relatively small needle hole. Emulsion characteristics were investigated on timepoints t = 0 h and t = 4 h. 

For the microscopic examination, 20 µL of the emulsion (after expulsion from the syringe and needle) was placed between two Menzel-Gläser cover slips (Schott AG, Mainz, Germany). Images were taken using a 20× magnification on an inverted transmitted light microscope (Leica Microsystems CMS GmbH, Mannheim, Germany). 

The emulsion stability was further analyzed by the water-drop test. Three drops of the prepared emulsions were dripped from the syringe via the needle into a test tube that contained 2 mL of demineralized water. The test was performed to investigate whether potential separation of the different emulsion phases could be observed [23]. 

In addition, differential light scattering (DLS) analysis was performed to characterize the particle size distribution and any potential changes over time [23]. One mL of the emulsion was expelled from the syringe coupled to the needle and the droplet size distribution was analyzed with a Wyatt Mobius (Wyatt Technology, Santa Barbara, CA, USA) differential light scattering apparatus equipped with a class I laser at 532.0 nm using a 163.5° scattering angle. The particle size distribution was reported as the 50% (Dv0.5) and 90% (Dv0.9) percentile.

### 2.8. Long-Term and In-Use Stability Study of the Peptide Mixtures

The long-term (i.e., frozen) and in-use (i.e., room temperature) stability of the four peptide mixture batches that were produced under GMP conditions were investigated according to the stability plan that is given in Supplementary Table S2. The release and end of shelf-life specifications are given in Supplementary Table S3.

Immediately after production and pulling the vials that were needed for the quality control analyses, the remaining vials were stored either at −20 to −30 °C (designated: −25 °C) or −70 to −90 °C (designated: −80 °C) in temperature-monitored freezers. The long-term stability was investigated for a storage period of 32 weeks. The stability samples stored at −80 °C were analyzed on timepoint t = 8 weeks and every timepoint after that. The sterility and bacterial endotoxins tests were only performed on the stability samples stored at −25 °C due to the limited amount of samples. For these tests, 8 vials (of the 32–34 vials) were needed according to the Ph. Eur. 2.6.1. The storage temperature of −25 °C was considered the worst-case scenario for the sterility and bacterial endotoxins tests since, theoretically, bacterial growth is less hampered at −25 °C compared to −80 °C and container closure integrity at −80 °C of the specific vial–stopper-seal combination was previously demonstrated. 

For the in-use stability study, peptide mixtures were analyzed immediately after thawing on the designated timepoints given in Supplementary Table S2, which corresponds to the t = 0 h results. Subsequently, the same samples were analyzed again after 2 h and 4 h stored at room temperature to investigate whether short-term storage at room temperature after thawing the samples had an effect on the stability of the peptide mixtures. 

## 3. Results

### 3.1. NOP Identification

In total, we analyzed 21 tumor biopsies from patients with NSCLC. Fifteen samples showed presence of frame-shift mutations of interest (71%). Additionally, we have analyzed tumor biopsies from other tumor types and the results of these analyses will be presented elsewhere (Martin et al., manuscript in preparation). In the present study, the framome of a lung tumor (LUN021) was selected as a model for generation of a synthetic long peptide vaccine. The framome was chosen because of its near-average size of 521 amino acids. 

**Figure 3:**
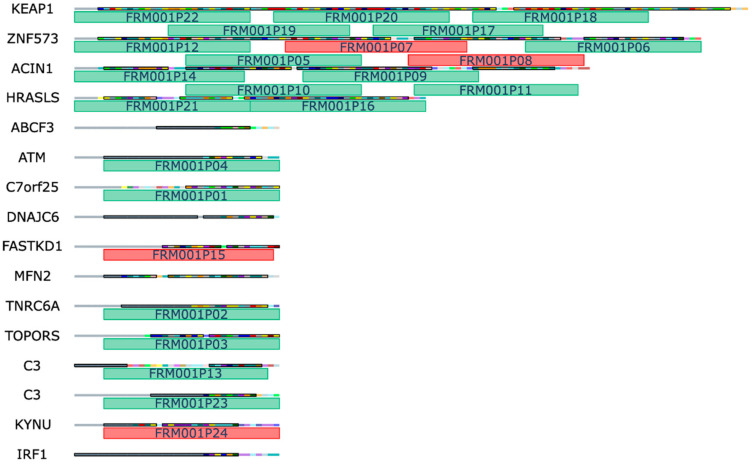
Peptide selection: Framome of lung tumor sample LUN021 where each row represents a NOP where multicolored squares are individual amino acids. Grey regions of the NOP represent appended upstream WT amino acids. Regions of NOP with predicted MHC binding epitopes are highlighted with a black box and enhanced color. The 24 selected peptides with indicated peptide IDs are shown as rectangular regions underneath their parent sequence, with green colored peptides being successfully manufactured while red peptides failed manufacturing (see also Section 3.2).

The framome of LUN021 consisted of 16 NOPs arising from 15 mutated genes with VAF and expression percentile listed in Table 2. A set of 24 peptides was selected from these NOPs as illustrated in Figure 3. Each peptide is designated with the identification code FRM001PX, where “X” is the peptide number. Peptide synthesis failed for the four peptides FRM001P07, FRM001P08, FRM001P15, and FRM001P24, as described in Section 3.2.

### 3.2. Peptide Synthesis

Twenty out of the 24 selected peptides (83%) were successfully synthesized at the required quantity and purity. Peptides were purified to ≥90% area purity with the required quantity set at >30 mg with the high throughput manufacturing process at a 0.25 mmol scale (Table 3). The crude peptides were obtained in yields ranging from 471–1139 mg (average 797 mg) and purities of between 12–84 area % (average 56 area %), whereas the purified gross weight ranged between 40–307 mg (average 186 mg) with corresponding purities of 92–99% (average 94%). The sequence of each synthesized peptide was 28–31 amino acid in length. The pI of the peptides ranged from 4–13. The spread of yield and crude purity reflects the diversity in sequence specific physical properties, which evidently exists even within a moderately small population of 24 peptide sequences.

The 20 peptides were formulated into four freeze-dried peptide mixtures that each contained six different peptides (FRM001-M1–FRM001-M4; see also Section 3.3). These four peptide mixtures were used for further processing.

### 3.3. Peptide Pool Design

**Figure 4:**
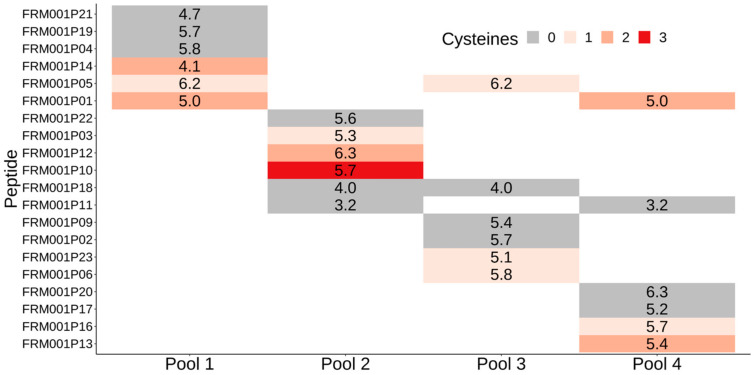
Peptide pooling scheme shows in each column one peptide pool where individual peptides are shown as rows. The color of each row corresponds to peptide cysteine content as depicted in the legend. The numbers shown in boxes indicate the peptide RT in seconds. Some peptides were added to multiple pools to achieve maximum of six peptides per pool and/or assure presence of three cysteine-containing peptides per each pool.

The successfully manufactured 20 peptides were pooled into four peptide mixtures each containing six peptides since the FRAME-001 can contain a maximum of four pools consisting of six peptides. The pools were made such that no peptide within a pool had measured retention times (RTs) within 0.2 s of each other to provide sufficient liquid chromatography resolution in downstream analysis. Cysteine residues can be of concern for peptide pooling due to the potential for dimer formation. To observe the effect of cysteine content on peptide pooling feasibility, pools were formed to ensure three out of six peptides contained at least one cysteine. Figure 4 shows the designed peptide mixtures with individual retention times measured by the peptide manufacturer. The peptide mixtures FRM001-M1, FRM001-M2, FRM001-M3, and FRM001-M4 corresponded to Pool 1, Pool 2, Pool 3, and Pool 4, respectively, in Figure 4.

### 3.4. Development and Evaluation of the Drug Product Production Process

A prospective risk assessment was performed in which several possible production processes and the associated risks were compared. The results showed that the most suited production process consisted of aseptically dissolving the peptide mixtures in an aqueous buffered solution, a subsequent sterile filtration step, and filling and capping of the primary containers. Possible risks associated with the production process that needed further assessment were the accuracy of the used syringes, potential adsorption of the peptides to the filter used for the sterile filtration step, aseptic process qualification, and the compatibility of the peptide mixture with Montanide for a stable emulsion that is suitable for subcutaneous injection.

According to the supplier, the syringe was ISO7886-1 certified and has a known accuracy of 96% (Becton Dickson Statement Volumetric Accuracy BD Syringes). The results of the accuracy test showed that the syringes had an accuracy of 99% with a standard deviation (SD) of 0.1 mL (*n* = 10), which confirmed the suitability of the syringe for the intended production process. 

Four representative batches of the peptide mixtures were produced under GMP conditions with the intended production process. The quality control analyses of the four peptide mixtures showed that the manufactured batches complied with the set requirements (Table 4, Table 5, Table 6 and Table 7). The aqueous peptide mixtures were sterile, pure, and had pH and osmotic values suitable for subcutaneous injection.

To investigate the effect of the filter used for the sterile filtration step, test samples before and after the filtration step were analyzed. The results showed no significant changes in peptide identity, peptide purity, peptide peak ratio, and retention times (Supplementary Tables S4–S6). Furthermore, no new impurities coming from the filter were observed, which demonstrates the suitability of the selected filter for the sterilization of the aqueous peptide mixtures. 

Finally, three consecutive TSB medium simulations were performed with the intended production process. After the 14-day incubation period of the medium, no microbial growth was observed. The growth promotion tests that followed immediately after the incubation period complied with the Ph. Eur. 2.6.1 and 2.6.12 requirements. Therefore, the production process of the peptide mixture was considered suitable and qualified at this stage of development.

### 3.5. Validation of the Stability-Indication Analytical Method

The objective of the stability-indicating analytical method was to demonstrate the suitability of the method for the intended purpose according to the ICH Q2 guideline [24]. The intended purpose of the analytical method was to assure a pure peptide mixture, i.e., to identify the main peptide peaks and potential impurities and degradation products. Challenges associated with the method validation were the lack of one given internal standard for the main peptides as well as impurities or degradation products since every peptide mixture is patient specific. Therefore, the constituents of the peptide mixtures differ from batch to batch. Taking this into consideration, three individual model peptides from the peptide mixture with different molecular characteristics (FRM001P02, FRM001P11, and FRM001P23), a stable isotope labeled reference standard, and the four peptide mixtures produced under GMP conditions were used to investigate the following validation parameters: system suitability, linearity (range), sensitivity, specificity, accuracy, precision, and the freeze–thaw stability. 

Table 8 shows the results of the analytical method validation. The analytical method was specific, sensitive, precise, accurate, and had a linear range between 5–225 µg/mL (3–157% normal sample loading). Furthermore, the linearity analysis showed that there was significant regression with no significant no lack-of-fit (data not shown). In addition, no individual point on the calibration curve deviated more than 10% (for the LLQ 15%) from the regression line. Finally, the results from the analysis of the aqueous peptide mixtures produced under GMP conditions demonstrated the suitable specificity of the method to detect the individual peptide peaks as well as potential impurities with an area ≥ 0.40%. Therefore, the analytical method was considered validated for the intended purpose at this stage of development.

### 3.6. Vaccine Emulsion Formulation and Stability

**Figure 5:**
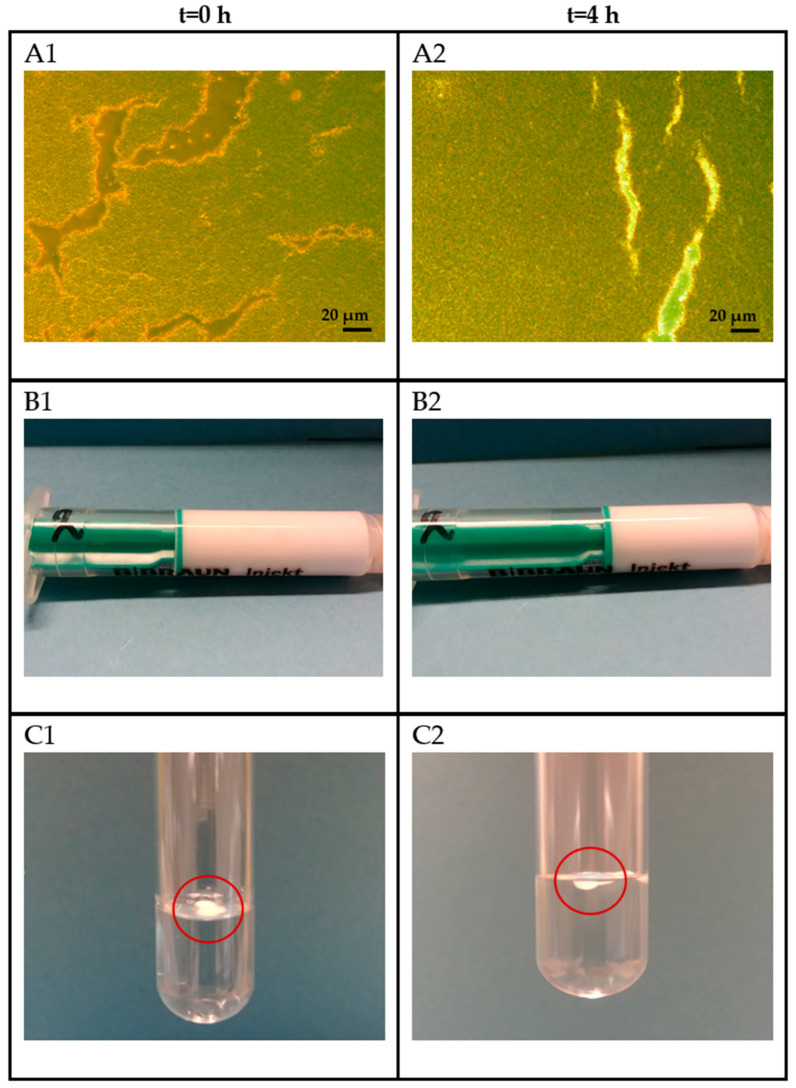
Representative images from the 4-h emulsion stability study at room temperature. (**A1**,**A2**): Microscopic examination at 20× magnification. (**B1**,**B2**): Visual inspection of the emulsion stored in the syringe. (**C1**,**C2**): The water-drop test to investigate potential phase separation of the water-in-oil emulsion. The red circle shows the emulsion droplet on the surface of the water without any emulsion phase separation.

The formulation buffer as well as the aqueous peptide mixtures were mixed and emulsified with Montanide. The stability of both emulsions was investigated for a 4 h period at room temperature. Table 9 gives the stability results and Figure 5 shows representative images from the stability study. 

The results show that the emulsion prepared either with the formulation buffer or peptide mixtures were stable for up to 4 h at room temperature. No phase separation was detected by visual, microscopic, and the water-drop test. Furthermore, no emulsion droplet coalescence could be observed since the emulsion droplet size did not increase over time. No difference between the test results of the emulsion prepared with the formulation buffer only or the aqueous peptide mixture were observed, indicating no significant effect of the neoantigen peptides on the emulsion stability at room temperature.

### 3.7. Long-Term and In-Use Stability Study of the Peptide Mixtures

The long-term stability of the four peptide mixtures manufactured under GMP conditions were investigated at the two storage conditions −25 °C or −80 °C for a storage period of 32 weeks (Supplementary Tables S2 and S3). For clarity, the stability results of only one peptide mixture (FRM001-M1) is shown in Table 10. The stability study results of the other three peptide mixtures were similar and complied with the set requirements. The complete stability data is given in Supplemental Tables S7–S9. 

The stability results show that the peptide identity, peptide purity, and appearance complied with the specification during the entire stability study. Furthermore, the sterility of the formulation was maintained during the entire storage period at the storage condition of −25 °C. The peptide peak ratio, pH, and osmolality did not change significantly over time. Small amounts of impurities were observed during the entire stability study. However, there was no clear trend in the formation of a given impurities or the amount of impurities. The end of shelf-life specification for the peptide purity (≥85%) was met for all formulations (lowest observed peptide purity: 88%). No significant changes in the chromatograms of the tested peptide mixtures on all time points were observed during the entire stability study (data not shown).

The in-use stability of the four peptide mixtures manufactured under GMP conditions were also investigated for a storage period of 32 weeks during the long-term stability study. The in-use stability of the peptide mixtures was analyzed immediately after thawing, and the samples were again analyzed after 2 h and 4 h stored at room temperature. No significant changes in peptide purity, peptide peak ratio, and impurities were observed (Supplementary Tables S10–S13). 

The results show that the four peptide mixtures were stable for a storage period of 32 weeks when stored either at −25 °C or −80 °C. In addition, the peptide mixtures were stable for up to 4 h at room temperature after thawing the samples on the designated time points during the 32-week stability study.

## 4. Discussion

FRAME-001 is a novel, personalized, neoantigen vaccine formulation that consists of up to four peptide mixtures that each can contain up to six patient-specific peptides. Patient-specific peptide selection was performed by analyzing lung tumor biopsy with WGS, short- and long-read RNA sequencing, and a novel, proprietary bioinformatics pipeline. Subsequently, the individual peptides were synthesized and analyzed with a flexible high throughput manufacturing platform to yield pure peptides that were suitable for further GMP processing. The manufactured peptide mixtures produced under GMP conditions were pure, stable, and met the pre-set requirements. Furthermore, the emulsion stability data showed that the formulation buffer as well as the four peptide mixtures yielded stable emulsions suitable for subcutaneous injection. The presented data show that FRAME-001 complied with GMP requirements, which makes it a feasible personalized vaccine formulation for the treatment of cancers with identified neoantigens.

Tumors containing mutated proteins have the potential to process and present the mutated short peptides in the context of MHC I molecules that can be recognized by cytotoxic T cells, leading to tumor cell destruction. Therapeutic cancer vaccines may potentially induce and/or strengthen the immune response towards such patient-specific tumor targets. However, personalized cancer vaccine design and manufacturing faces unique challenges as each drug product is individually prepared for each patient, specifically tailored to their tumor profile.

The initial step in the production process of a personalized cancer vaccine is identifying and selecting the neoantigens within the tumor with the highest probability of inducing a strong immune response against the tumor cells. Many previously tested neoantigen-based cancer vaccines contained peptide sequences selected from most common point mutations, where each neoantigen peptide sequence differed from its wild-type counterpart by only one amino acid. We have developed a method based on a combination of WGS with short- and long-RNA sequencing, followed by bioinformatics pipeline that together allow identification and selection of neoantigens formed by completely novel stretches of amino acids. Applying this method, we were able to identify and select so-called NOPs with high potential to generate multiple immunogenic epitopes across several HLA alleles. 

The selection of the peptides that are most suitable for the production of the polypeptide cancer vaccine is the next important step. The algorithm we developed to assist in further selection of the peptides and their combination in mixtures, can evaluate several relevant parameters, such as predicted binding affinity of epitopes to various HLA alleles, hydrophobicity, and cysteine content of the peptides. The latter is of importance since the cysteine content of the peptides may have substantial consequences for the stability of the formulation.

For the patient sample tumor LUN021 that we used as a model for this study, we have observed that significant number of selected peptides contained at least one cysteine. The exclusion of all peptides with cysteine would dramatically decrease the number of peptides suitable for the final vaccine composition, which may negatively impact the desired overall anti-tumor immune response. In previously published clinical trials, seemingly various approaches have been used concerning cysteine-containing peptides. The study of Hilf et al. [25] reports that none of the investigated twenty peptides contained a cysteine, which suggests that cysteine-containing peptides have been avoided. In two other clinical studies published by Ott et al. [11,12], the number of peptides containing at least one cysteine was reduced to 11% and 12%, with the number of peptides containing more than two cysteines being only 1.3% and 0.4%, respectively. Only one peptide out of more than 1000 peptides used in total in both studies had a cysteine at the N- or C-terminus, which again suggests efforts in reducing the number of potentially problematic cysteine-containing sequences in the final vaccine formulation. 

On the contrary, the study of Melief et al. [26] using peptides derived from human papillomavirus (HPV) oncogenic virus proteins E6 and E7 had all peptide sequences containing at least one cysteine, although none of them was either present at the N- or C-terminus. Similarly, in the study of Fang et al. [27], no obvious avoidance of cysteines in the peptide sequences was observed as almost half (43%) of all used peptides contained at least one cysteine. This shows that there is no clear consensus in the field of personalized peptide cancer vaccines on whether the exclusion of cysteines is necessary. 

One can speculate that such decision during peptide sequence selection may depend on the number of mutated peptide sequences that are suitable candidates for a vaccine. The studies performed by Hilf [25] and Ott [11,12] used predominantly peptides containing point mutations, which are usually present in high numbers in some tumors. The selection based on cysteine content may therefore be performed without compromising the vaccine content. In our study, inclusion of such a step would decrease the number of potentially highly immunogenic neoantigen sequences. Instead of excluding cysteine-containing sequences from our selection, we opted to manufacture the GMP batches with a maximum of three cysteine-containing peptides per mixture to simulate the worst-case scenario for the stability study. 

The manufacturing process of freeze-dried peptide mixtures (drug substance) for personalized cancer vaccines requires rapid, GMP compliant, and cost-effective peptide synthesis. This was achieved by utilizing a high throughput manufacturing and analysis platform rather than a product-specific process. The FRAME-001 platform achieved an initial manufacturing speed of 20 days from initiation to release of the individual peptides. Further refinement of the platform achieved a manufacturing speed of 15 days. The peptide synthesis results demonstrated that the platform was highly robust, had an acceptable low failure level (17%), and was feasible to produce personalized peptide mixtures.

Since the physical properties of peptides can vary in extremes from one sequence to another, any high throughput platform will have applicability limits across a peptide set. Consequently, there will be a certain level of manufacturing failure within acceptable limit. The four peptides not included in the peptide mixtures could not be purified to a purity >90% area, and thus, were deemed incompatible with the platform process. The 20 synthesized peptides were formulated into four different freeze-dried peptide mixtures that each contained six peptides for further GMP processing. 

The risk assessment for the production process of the aqueous peptide mixtures (drug product) showed that there were several risks that needed further investigation. The associated risks were the volumetric accuracy of the syringe, peptide adsorption to the sterilization filter, the sterility of the formulation, the solubility and stability of the peptides in the formulation medium, and the compatibility of the formulation with Montanide for a stable emulsion that is suitable for subcutaneous injection. 

The accuracy of the syringe stated by the supplier was 4%, which was considered acceptable. Our own accuracy test showed that the syringe had an accuracy of 1% with a low standard deviation. The syringe is sterile, cheap, widely available, and single use only. Furthermore, it is compatible with a wide range of single-use sterilization filters. However, peptide adsorption to the sterilization filter that was used during the production process was a potential risk. 

A variety of factors are involved in protein adsorption to filters, such as the hydrophilicity/hydrophobicity of the filter surface, the constituents of the filtered solvent (ionic strength, excipients, and pH), electrostatic charges of the filter surface, and the inherent protein characteristics such as charges and three-dimensional structure [28,29]. Even though these phenomena may be less relevant for peptides, a hydrophilic polyvinylidene fluoride (PVDF) filter that is known for a low protein adsorption was chosen. The peptide mixture analysis before and after the sterile filtration step showed that there was no significant filter effect. Therefore, the investigated filter was considered suitable for the sterilization step of the aqueous peptide mixtures. 

The optimal formulation medium should comply with the following requirements. First, it needs to be safe and suitable for subcutaneous injection. Second, a wide range of peptides should be soluble and stable in the solution. Third, it should mix and emulsify readily with the adjuvant Montanide to form a stable emulsion at room temperature. 

Generally speaking, peptide solubility and stability issues may be resolved by modifying the pH, ionic strength, or polarity of the solution (i.e., adding organic solvents such as dimethyl sulfoxide [DMSO] or surfactants) [30]. From a safety and regulatory point of view, the formulation medium should be standardized and have no toxic potential. Subcutaneous injection of a solution that has a pH that substantially deviates from 6–8 might cause painful irritation at the injection site [31]. Furthermore, solutions containing a substantial amount of organic solvents such as DMSO have toxic potential and may not readily form a stable emulsion with Montanide. The latter may also be true for solutions containing substantial amounts of surfactants [32]. Additionally, pH shifts in unbuffered solutions may result in a pH ≠ 6–8 or in peptide degradation, aggregation, or precipitation [33]. Considering all of the above, we opted for PBS as the formulation medium since this was a sterile and low-endotoxin formulation with a pH = 7.0, which is intended to be used as a parenteral formulation. 

The peptide mixtures were readily soluble in the chosen formulation buffer, even though the physiochemical properties of the individual peptides differed greatly. Major challenges associated with the long-term stability of peptides in solution are the oxidation of aromatic or sulfur-containing amino acid residues (histidine, tryptophan, tyrosine, methionine, and cysteine), the formation of pyro-glutamate from N-terminal glutamine, deamination of asparagine and glutamine residues, peptide multimerization through cysteine residue sulfur bonds, and the hydrolysis of the peptide bond, specifically aspartic acid (D)–proline (P) (D-P) cleavage [30,33,34,35,36]. These modifications in the molecular structure not only yield impurities, but may also affect the antigenicity of the peptides [37].

Acidic and alkaline conditions are known to promote deamination, D-P cleavage, oxidation, and glutamine cyclization, whereas exposure to light, oxygen, and high storage temperatures potentially increase the oxidation of peptide residues [30,33,34,35,36]. It was therefore expected that the neutral pH of the formulation buffer as well as the storage conditions at low temperatures and protected from light were beneficial for the peptide stability. The results of the long-term (i.e., frozen) and in-use (i.e., room temperature) stability study indeed showed that the four peptide mixtures remained stable for up to 32 weeks stored at both storage conditions. Except for peptide dimer formation through cysteine sulfur bonds, no significant amounts (≥5%) of one given peptide impurity or degradation product was observed. 

Cysteine-containing peptide formulations have been reported to form dimers (homo- and heterodimers), especially at relatively higher storage temperatures [34,36]. At the end of shelf life, 0–9% and 0–10% of dimers were observed at the storage condition −25 °C and −80 °C, respectively, which was considered acceptable for a 32-week storage period. We did not categorize the formation of dimers that formed by disulfide bridges between the thiol groups of cysteine residues as an impurity since this is a frequent phenomenon amongst natural proteins. This approach is justified given the balance between the in vivo monomeric and dimeric states which is known to occur for cysteine containing peptides. Furthermore, an observed dimer in vitro does not mean that the biological activity of the dimer is negatively affected and the dimer also remains in vivo [38,39,40]. It is well known that cysteine-containing peptides represent a large portion of the immuno-peptidome presented on MHC molecules at the cell surface and that such peptides can elicit strong immune responses [36]. We thus considered the presence of cysteine-containing peptides essential for FRAME-001. However, we cannot completely disregard that the presence of cysteines might negatively influence the biological activity of the vaccine. The correlation between the vaccine immune response and peptide cysteine content will indeed be performed as part of the efficacy analysis in the clinical trial.

The peptide mixtures were mixed with the vaccine adjuvant Montanide to yield a water-in-oil emulsion. Antigen depot formation after the emulsion is injected is considered as a mechanism of action of the adjuvant. The antigen depot does not only protects the antigens from degradation, but also releases the antigens in a sustained manner from the emulsion droplets [18,19]. Therefore, a stable vaccine emulsion is essential for the effectiveness of FRAME-001. For the emulsion stability study, the neoantigen vaccine emulsion was stored in the primary container that is used for the subcutaneous injection, i.e., B. Braun Injekt syringe with a 25G needle. Stable emulsions were readily formed by the two-syringe method proposed by the supplier (Seppic S.A., La Garenne-Colombes, France) when the Montanide was either emulsified with the formulation buffer only or the aqueous peptide mixtures. No difference between the stability results of the formulation buffer only or aqueous peptide mixtures were observed, which showed the suitability of Montanide as a vaccine adjuvant for FRAME-001.

For the stability-indicating analytical method of the peptide mixtures, system suitability, linearity (range), sensitivity, specificity, accuracy, precision, and the freeze–thaw stability were demonstrated according to the ICHQ2 guideline [24]. The main challenges associated with the validation of the analytical method were the lack of an internal standard and one specific reference peptide mixture and impurity that covers all future patient-specific batches. The constituents as well as the potentially present impurities of FRAME-001 differ due to the personalized nature of the formulation. For the internal standard, one strategy was to add the standard to the peptide mixtures during the production process or analytical sample preparation. However, incompatibility issues with the investigated as well as future peptide mixtures may render this approach not feasible. Furthermore, the analytical method was developed in view of ensuring a pure peptide mixture and this objective does not necessitate the use of an internal standard.

The objective of the validation strategy was to demonstrate that the method was suitable for the analysis of representative peptide mixtures and potentially present impurities that correspond to ≥0.40% of the total peak area. The method had a linear range of 3%–157% normal sample loading, was suitable for the analysis of a wide range of peptides that had different physiochemical characteristics and was sensitive for the analysis of the peptides (signal-to-noise ratio range: 45–160) as well as impurities. The method could also differentiate between different entities co-eluted at one chromatogram peak (data not shown). Considering all of the above, the analytical method was considered validated during this stage of development. The analytical results of future peptide mixtures will be used to substantiate the validation status of the method.

Due to the personalized nature of FRAME-001, a possibility exists that certain peptides that are synthesized for a given patient may not be stable or readily dissolve in the formulation buffer. These peptides could be identified during the quality control analysis and the following stability study of the manufactured peptide mixtures. Subsequently, peptide characteristics studies should identify peptide-specific properties that result in peptide solubility and stability issues. Moreover, the present study primarily focused on the development and feasibility of FRAME-001. No immunological studies on the selected neoantigen peptides were performed as part of the study. The objectives of the FRAME-001 phase II clinical trial are to assess the immunogenicity, safety, tolerability, and clinical efficacy of the formulation. The immunogenicity may in turn be correlated to the selected peptide and neoantigen characteristics. These data may provide information for future peptide selection and process optimization.

## 5. Conclusions

FRAME-001 is a novel, personalized, neoantigen vaccine formulation that complied with GMP requirement. Neoantigen peptide selection was performed by genetic analysis of lung tumor biopsy material combined with a novel bioinformatics pipeline. The selected peptides were synthesized with a flexible high throughput platform and further formulated into stable aqueous peptide mixtures that formed stable emulsion with the vaccine adjuvant Montanide. The presented data show that FRAME-001 is a feasible personalized vaccine formulation for the treatment of stage III-IV NSCLC. To the best of our knowledge, even though neoantigen peptides have been investigated in multiple clinical trials, the literature does not describe a strategy for the development of a personalized neoantigen vaccine formulation for clinical use. Therefore, the presented strategy may give guidance in the development of novel personalized peptide therapeutic cancer vaccines.

## 6. Patents

European patent application No. 20160013.7 and 20184218.4 (framome-based vaccine) were filed describing technology for detection of Frame neoantigens using FramePro technology, use of framome as target of cancer vaccines, and the use of hidden Frames as immunotherapy target.

## Figures and Tables

**Table 1:** The specifications and acceptance criteria of the peptide mixtures that will be formulated together with the adjuvant Montanide into FRAME-001.

Specifications		Acceptance Criteria
Peptide identity	Peptide 1	[M+H]^+^ monoisotopic expected mass ± 2 atomic mass units (amu)
	Peptide 2	
	Peptide 3	
	Peptide 4	
	Peptide 5	
	Peptide 6	
Peptide peak ratio ^1^	Peptide 1	Report result
	Peptide 2	
	Peptide 3	
	Peptide 4	
	Peptide 5	
	Peptide 6	
Peptide purity ^2^		≥85% of total area
Impurities (%) ^3^		Report result
Bacterial endotoxins ^4^		<20 EU/mL
Sterility		Sterile
Appearance		Clear colorless solution
pH		6–8
Osmolality		270–310 mOsm/kg
Fill volume		1.40 mL
Emulsification		0.90 mL peptide mixture + 0.90 mL Montanide
Emulsion stability		Forms stable emulsion with Montanide at room temperature for at least 4 h
Route of administration		Subcutaneously, 1.40 mL neoantigen vaccine emulsion
Long-term stability		At least 9 weeks
In-use stability		At least 4 h immediately after thawing

^1^ Peptide peak ratio is reported as the % area of the individual peptide chromatogram peak in relation to the sum of all peak areas of the individual peptides (=100%). ^2^ Peptide purity is the sum of the main peptide peak areas divided by the total observed area on the chromatogram of the test sample. ^3^ Any observed chromatogram peak area of ≥0.40% not corresponding to the main peptide peak areas. ^4^ Based on Ph. Eur. 5.1.10.

**Table 2:** Purity corrected VAFs and expression percentiles for genes leading to a NOP in lung tumor sample LUN021.

Gene	VAF	Expression Percentile
KEAP1	0.79	0.51
ZNF573	0.71	0.70
ACIN1	0.41	0.24
HRASLS	0.87	0.53
ABCF3	1.08	0.81
ATM	0.39	0.97
C7orf25	0.59	0.74
DNAJC6	0.44	0.63
FASTKD1	0.75	0.67
MFN2	0.29	0.30
TNRC6A	0.84	0.93
TOPORS	0.43	0.78
C3	0.23	0.39
KYNU	0.31	0.19
IRF1	0.13	0.53

**Table 3:** The results of the peptide synthesis and freeze-dried peptide mixtures. The synthesized peptides had a sequence of 28–31 amino acids.

Peptide	Purified Gross Weight (mg)	Purity (Area %)	pI	Peptide Mixture	Gross Quantity Added to Peptide Mixture (mg)	Peptide Mixture Purity (Area %)
FRM001P01	183 ^1^	93 ^1^	12	FRM001-M1	31	94
FRM001P04	113	94	11		28	
FRM001P05	280 ^1^	96 ^1^	11		27	
FRM001P14	190	94	8		28	
FRM001P19	199	94	12		28	
FRM001P21	40	99	13		22	
FRM001P03	55	97	5	FRM001-M2	27	95
FRM001P10	84	93	4		26	
FRM001P11	269 ^1^	95 ^1^	13		31	
FRM001P12	227	95	10		28	
FRM001P18	270 ^1^	93 ^1^	6		28	
FRM001P22	207	95	13		23	
FRM001P02	263	95	11	FRM001-M3	29	95
FRM001P05	280 ^1^	96 ^1^	11		27	
FRM001P06	147	94	10		27	
FRM001P09	57	92	10		28	
FRM001P18	270 ^1^	93 ^1^	6		28	
FRM001P23	243	92	4		24	
FRM001P01	183 ^1^	93 ^1^	12	FRM001-M4	31	93
FRM001P11	269 ^1^	95 ^1^	13		31	
FRM001P13	123	93	9		28	
FRM001P16	174	93	7		28	
FRM001P17	307	97	8		26	
FRM001P20	291	95	4		26	
FRM001P07	Peptides could not be successfully synthesized					
FRM001P08						
FRM001P15						
FRM001P24						

^1^ These peptides were synthesized only once but used to formulate two freeze-dried peptide mixtures.

**Table 4:** The tests results of peptide mixture FRM001-M1 that was manufactured under GMP conditions.

Test		Specification	Result
Peptide identity (amu)	FRM001P01	3625.9 ± 2	3626.9
	FRM001P04	3439.9 ± 2	3439.9
	FRM001P05	3559.0 ± 2	3560.0
	FRM001P14	3103.4 ± 2	3103.4
	FRM001P19	3249.9 ± 2	3249.9
	FRM001P21	3521.9 ± 2	3521.9
Peptide peak ratio (%)	FRM001P01	Report result	15.0
	FRM001P04		16.1
	FRM001P05		25.2
	FRM001P14		10.8
	FRM001P19		23.1
	FRM001P21		9.8
Peptide purity		≥85% area	98%
Impurities (%) ^1^		Report result	0.47 (RRT 0.16)
Bacterial endotoxins		<20 EU/mL	<0.5 EU/mL
Sterility		Sterile	Sterile
Appearance		Clear colorless solution	Clear colorless solution
pH		6–8	7
Osmolality		270–310 mOsm/kg	293 mOsm/kg

^1^ Result is reported as relative retention time relative (RRT) to FRM001P14 (first eluting peptide) and % area of the total sum of areas if the impurity peak area is ≥0.40%.

**Table 5:** The tests results of peptide mixture FRM001-M2 that was manufactured under GMP conditions.

Test		Specification	Result
Peptide identity (amu)	FRM001P03	3425.7 ± 2 u	3425.7
	FRM001P10	3320.5 ± 2 u	3320.5
	FRM001P11	3231.7 ± 2 u	3231.8
	FRM001P12	3475.9 ± 2 u	3475.9
	FRM001P18	2967.5 ± 2 u	2967.5
	FRM001P22	3450.0 ± 2 u	3450.1
Peptide peak ratio (%)	FRM001P03	Report result	9.2
	FRM001P10		9.1
	FRM001P11		13.7
	FRM001P12		24.6
	FRM001P18		21.4
	FRM001P22		22.1
Peptide purity		≥85% area	95%
Impurities (%) ^1^		Report result	0.76 (RRT 0.24) 0.47 (RRT 2.91)
Bacterial endotoxins		<20 EU/mL	<0.5 EU/mL
Sterility		Sterile	Sterile
Appearance		Clear colorless solution	Clear colorless solution
pH		6–8	7
Osmolality		270–310 mOsm/kg	296 mOsm/kg

^1^ Result is reported as retention time relative (RRT) to FRM001P11 (first eluting peptide) and % area of the total sum of areas if the impurity peak area is ≥0.40%.

**Table 6:** The tests results of peptide mixture FRM001-M3 that was manufactured under GMP conditions.

Test		Specification	Result
Peptide identity (amu)	FRM001P02	3432.9 ± 2 u	3434.0
	FRM001P05	3559.0 ± 2 u	3559.9
	FRM001P06	3268.7 ± 2 u	3268.7
	FRM001P09	3380.6 ± 2 u	3380.6
	FRM001P18	2967.5 ± 2 u	2967.2
	FRM001P23	3329.6 ± 2 u	3329.6
Peptide peak ratio (%)	FRM001P02	Report result	18.7
	FRM001P05		22.7
	FRM001P06		16.4
	FRM001P09		14.2
	FRM001P18		16.2
	FRM001P23		11.8
Peptide purity		≥85% area	97%
Impurities (%) ^1^		Report result	0.45 (RRT 1.65)
Bacterial endotoxins		<20 EU/mL	<0.5 EU/mL
Sterility		Sterile	Sterile
Appearance		Clear colorless solution	Clear colorless solution
pH		6–8	7
Osmolality		270–310 mOsm/kg	293 mOsm/kg

^1^ Result is reported as retention time relative (RRT) to FRM001P18 (first eluting peptide) and % area of the total sum of areas if the impurity peak area is ≥0.40%.

**Table 7:** The tests results of peptide mixture FRM001-M4 that was manufactured under GMP conditions.

Test		Specification	Result
Peptide identity (amu)	FRM001P01	3625.9 ± 2 u	3625.8
	FRM001P11	3231.7 ± 2 u	3231.5
	FRM001P13	3235.6 ± 2 u	3235.4
	FRM001P16	3545.8 ± 2 u	3545.7
	FRM001P17	3003.6 ± 2 u	3003.4
	FRM001P20	3037.7 ± 2 u	3037.9
Peptide peak ratio (%)	FRM001P01	Report result	14.4
	FRM001P11		11.5
	FRM001P13		14.2
	FRM001P16		13.6
	FRM001P17		20.3
	FRM001P20		25.7
Peptide purity		≥85% area	97%
Impurities (%) ^1^		Report result	0.43 (RRT 0.23)
Bacterial endotoxins		<20 EU/mL	<0.5 EU/mL
Sterility		Sterile	Sterile
Appearance		Clear colorless solution	Clear colorless solution
pH		6–8	7
Osmolality		270–310 mOsm/kg	294 mOsm/kg

^1^ Result is reported as retention time relative (RRT) to FRM001P11 (first eluting peptide) and % area of the total sum of areas if the impurity peak area is ≥0.40%.

**Table 8:** The validation results of the stability-indicating LC-MS analytical method. FRM001P02, FRM001P11, and FRM001P23 are designated P02, P11, and P23, respectively.

Test	Acceptance Criteria	Results						
System suitability, reference	Within day RSD: ≤3.0% for 100 µg/mL	Range: 0.3–0.8%						
System suitability, model peptides	Within day RSD: ≤3.0% for 50 µg/mL, 150 µg/mL, and 225 µg/mL	P02 (range): 0.3–1.9% P11 (range): 0.6–2.6% P23 (range): 0.9–2.5%						
Linearity	R^2^ ≥ 0.99	P02: 1.00 P11: 1.00 P23: 1.00						
Range	5–225 µg/mL (3–157% normal sample loading)	P02: 5–225 µg/mL P11: 5–225 µg/mL P23: 5–225 µg/mL						
Accuracy and Precision	Bias and RSD: ≤10% Bias and RSD LOQ: ≤15%		LOQ		150 µg/mL		225 µg/mL	
		Peptide	Bias (%)	RSD (%)	Bias (%)	RSD (%)	Bias (%)	RSD (%)
		P02	2	9	10	3	2	4
		P11	13	14	0	2	4	3
		P23	9	1	2	2	0	1
Sensitivity (LOQ)	Signal-to-noise ratio ≥ 10	P02: S/N ratio 160 P11: S/N ratio 145 P23: S/N ratio 45						
Freeze–thaw stability	Average bias: ≤10% Within run RSD: ≤10% Between run RSD: ≤10%	Peptide		Average bias (%)		Within run RSD (%)	Between run RSD (%)	
		P02		8		1	4	
		P11		1		1	3	
		P23		4		1	3	
Specificity model peptides and reference	[M + H]^+^ monoisotopic expected mass ± 2 amu	Conform for the reference standard and three model peptides at the different peptide concentrations on all three consecutive days						
Specificity GMP peptide mixtures	[M + H]^+^ monoisotopic expected mass ± 2 amu	Complies for the four peptide mixtures. See also Table 4, Table 5, Table 6 and Table 7.						
Impurities in GMP peptide mixtures	Areas ≥ 0.40% not corresponding to the six main peptide peaks of the peptide mixture are clearly detectable	Complies for the four peptide mixtures. See also Table 4, Table 5, Table 6 and Table 7.						

**Table 9:** The emulsion stability study results. Emulsions were prepared either with the formulation buffer only or the aqueous peptide mixtures that were manufactured under GMP conditions. Analyses were performed in triplicate.

Test	Solution	Specification	Objective	Result t = 0 h	Result t = 4 h
Water-drop test	Formulation buffer	Emulsion droplets do not combine with the water for 5 s	Identification of phase separation	Conform	Conform
	Peptide mixture			Conform	Conform
Microscopic examination	Formulation buffer	Homogenous distribution of the emulsion droplets with no observable phase separation	Identification of phase separation	Conform	Conform
	Peptide mixture			Conform	Conform
Differential light scattering	Formulation buffer	Report DV0.5 and DV0.9 results	Emulsion particle size distribution	DV0.5: 0.25 ± 0.06 µm DV0.9: 0.37 ± 0.10 µm	DV0.5: 0.20 ± 0.05 µm DV0.9: 0.38 ± 0.09 µm
	Peptide mixture			DV0.5: 0.19 ± 0.07 µm DV0.9: 0.33 ± 0.13 µm	DV0.5: 0.15 ± 0.09 µm DV0.9: 0.29 ± 0.08 µm

**Table 10:** The stability results of the FRM001-M1 peptide mixture stored at −25°C and −80°C.

Part 1/2									
Test		Specification	t = 0 w	t = 2 w −25 °C	t = 4 w −25 °C	T = 8 w −25 °C	t = 8 w −80 °C	t = 12 w −25 °C	t = 12 w −80 °C
Identity	FRM001P01	3625.9 ± 2 u	3626.9	3626.9	3623.9	3626.9	3626.9	3626.9	3626.9
	FRM001P04	3439.9 ± 2 u	3439.9	3439.9	3439.9	3439.9	3439.9	3439.9	3439.9
	FRM001P05	3559.0 ± 2 u	3560.0	3560.0	3560.0	3560.0	3560.0	3560.0	3560.0
	FRM001P14	3103.4 ± 2 u	3103.4	3103.4	3103.1	3103.4	3103.4	3103.4	3103.4
	FRM001P19	3249.9 ± 2 u	3249.9	3249.9	3249.9	3249.9	3249.9	3249.9	3249.9
	FRM001P21	3521.9 ± 2 u	3521.9	3521.9	3521.9	3521.9	3521.9	3521.9	3521.9
Peak ratio (%)	FRM001P01	Report result	15.0	15.0	14.6	15.2	15.0	15.9	15.5
	FRM001P04	Report result	16.1	15.7	16.7	16.7	16.6	16.4	17.3
	FRM001P05	Report result	25.2	25.1	25.4	25.3	25.3	24.5	26.8
	FRM001P14	Report result	10.8	11.2	10.5	10.9	10.8	11.9	7.9
	FRM001P19	Report result	23.1	22.8	22.1	21.3	21.7	19.9	21.6
	FRM001P21	Report result	9.8	10.2	10.7	10.6	10.7	11.3	11.1
Peptide purity		≥85% area	98%	98%	95%	94%	97%	94%	98%
Impurities (%) ^1^		Report result	0.47 (RRT 0.16)	0.41 (RRT1.70)	0.62 (RRT 2.60) 0.67 (RRT 2.71) 0.51 (RRT 2.79)	0.46 (RRT 0.15) 0.65 (RRT 2.39) 0.41 (RRT 2.43) 0.63 (RRT 2.60) 0.60 (RRT 2.63) 0.58 (RRT 2.70) 0.48 (RRT 2.76)	0.53 (RRT 0.17) 0.48 (RRT 2.58) 0.51 (RRT 2.72)	0.51 (RRT 2.51) 0.69 (RRT 2.57) 0.46 (RRT 2.62) 1.60 (RRT 2.73)	0.44 (RRT 0.16)
Bacterial Endotoxins		<20 EU/mL	<0.5 EU/mL	n/a	n/a	n/a	n/a	n/a	n/a
Sterility		Sterile	Sterile	n/a	n/a	n/a	n/a	n/a	n/a
Appearance		Clear colorless solution	Clear color- less solution	Clear colorless solution	Clear colorless solution	Clear colorless solution	Clear colorless solution	Clear colorless solution	Clear colorless solution
pH		6–8	7	n/a	n/a	7	7	7	7
Osmolality (mOsm/kg)		270–310 mOsm/kg	293	n/a	n/a	296	298	294	292
**Part 2/2**									
**Test**		**Specification**		**t = 0 w**	**t = 16 w −25 °C**		**t = 16 w −80 °C**	**t = 32 w −25 °C**	**t = 32 w −80 °C**
Identity	FRM001P01	3625.9 ± 2 u		3626.9	3626.9		3626.9	3626.9	3626.9
	FRM001P04	3439.9 ± 2 u		3439.9	3439.9		3439.9	3439.9	3439.9
	FRM001P05	3559.0 ± 2 u		3560.0	3560.0		3560.0	3560.0	3560.0
	FRM001P14	3103.4 ± 2 u		3103.4	3103.4		3103.4	3103.4	3103.4
	FRM001P19	3249.9 ± 2 u		3249.9	3249.9		3249.9	3249.9	3249.9
	FRM001P21	3521.9 ± 2 u		3521.9	3521.9		3521.9	3521.9	3521.9
Peak ratio (%)	FRM001P01	Report result		15.0	15.4		15.3	13.4	14.2
	FRM001P04	Report result		16.1	17.2		16.6	18.4	16.7
	FRM001P05	Report result		25.2	25.6		25.7	26.4	24.9
	FRM001P14	Report result		10.8	10.1		10.7	7.5	11.8
	FRM001P19	Report result		23.1	21.5		21.4	22.8	21.4
	FRM001P21	Report result		9.8	10.1		10.3	11.5	11.1
Peptide purity			≥85% area	98%	97%		97%	92%	95%
Impurities (%) ^1^			Report result	0.47 (RRT 0.16)	0.40 (RRT 2.58) 0.61 (RRT 2.73)		0.52 (RRT 2.63)	0.81 (RTT 2.74) 0.55 (RRT 2.79) 0.88 (RRT 2.84) 1.12 (RRT 2.98) 2.09 (RRT 3.01) 1.11 (RRT 3.12) 0.92 (RRT 3.16) 0.53 (RRT 3.21)	0.44 (RRT 2.96) 0.78 (RRT 3.12) 0.65 (RRT 3.14) 0.56 (RRT 3.16)
Bacterial Endotoxins			<20 EU/mL	<0.5 EU/mL	n/a		n/a	<0.5 EU/mL	n/a
Sterility			Sterile	Sterile	297		295	Sterile	n/a
Appearance			Clear colorless solution	Clear color- less solution	Clear colorless solution		Clear colorless solution	Clear colorless solution	Clear colorless solution
pH			6–8	7	7		7	7	7
Osmolality (mOsm/kg)			270–310 mOsm/kg	293	297		295	295	285

^1^ Result is reported as retention time relative (RRT) to FRM001P14 (first eluting peptide) and % area of the total sum of areas if the impurity peak area is ≥0.40%. Abbreviation n/a: not applicable.

## Data Availability

Data is contained within the article and Supplementary Materials.

## Supplementary Materials

The following supporting information can be downloaded at: https://www.mdpi.com/article/10.3390/pharmaceutics14071515/s1, Figure S1: Schematic representation of the peptide and peptide mixtures manufacturing platform; Figure S2: The appearance of the emulsion that was formed by the two-syringe and i-connector method according to the instructions of the supplier (Seppic S.A., France); Table S1: The characteristics of the 20 different neoantigen peptides; Table S2: The long-term stability plan of the peptide mixtures that were produced under GMP conditions; Table S3: The release, in-use, and end of shelf life specifications for the stability study of the peptide mixtures; Table S4: The results of the filter experiment. Unfiltered and filtered test samples of the peptide mixture batches FRM001-M1, FRM001-M2, FRM001-M3, and FRM001-M4 were analyzed to investigate potential filter effects; Table S5: The chromatograms of the filtered and unfiltered test samples of the peptide mixtures FRM001-M1 and FRM001-M2; Table S6: The chromatograms of the filtered and unfiltered test samples of the peptide mixtures FRM001-M3 and FRM001-M4; Table S7: The stability results of the FRM001-M2 peptide mixture stored at −25 °C or −80 °C; Table S8: The stability results of the FRM001-M3 peptide mixture stored at −25 °C and −80 °C; Table S9: The stability results of the FRM001-M4 peptide mixture stored at −25 °C and −80 °C; Table S10: The results of the in-use stability of the FRM001-M1 batch. After the t = 0 h analysis, the same sample was stored at room temperature and analyzed again after 2 h and 4 h. For readability, only the 0 h and 4 h data are presented; all 2 h data were similar and complied; Table S11: The results of the in-use stability of the FRM001-M2 batch. After the t = 0 h analysis, the same sample was stored at room temperature and analyzed again after 2 h and 4 h. For readability, only the 0 h and 4 h data are presented; all 2 h data were similar and complied; Table S12: The results of the in-use stability of the FRM001-M3 batch. After the t = 0 h analysis, the same sample was stored at room temperature and analyzed again after 2 h and 4 h. For readability, only the 0 h and 4 h data are presented; all 2 h data were similar and complied; Table S13: The results of the in-use stability of the FRM001-M4 batch. After the t = 0 h analysis, the same sample was stored at room temperature and analyzed again after 2 h and 4 h. For readability, only the 0 h and 4 h data are presented; all 2 h data were similar and complied.
