# Supplementary material for: Development of a Personalized Tumor Neoantigen Based Vaccine Formulation (FRAME-001) for Use in a Phase II Trial for the Treatment of Advanced Non-Small Cell Lung Cancer
Source: Pharmaceutics. 2022 Jul 21;14(7):1515. doi: 10.3390/pharmaceutics14071515 (PMC9322189; doi:10.3390/pharmaceutics14071515)
Supplement: Supplementary file 1 [file pharmaceutics-14-01515-s001.zip › pharmaceutics-1802305-supplementary.pdf]

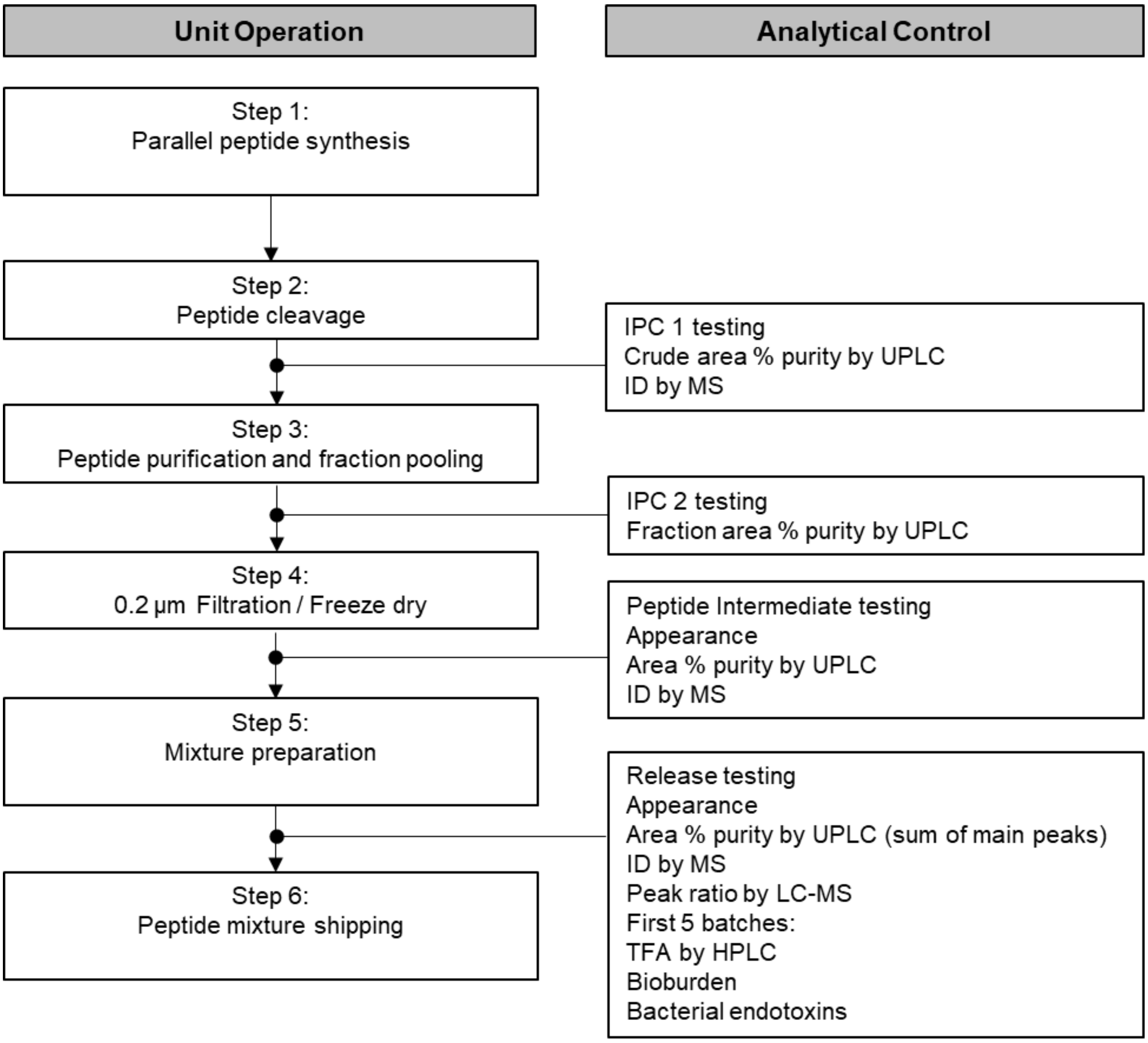

Supplementary Figure S1. Schematic representation of the peptide and peptide mixtures manufacturing platform.

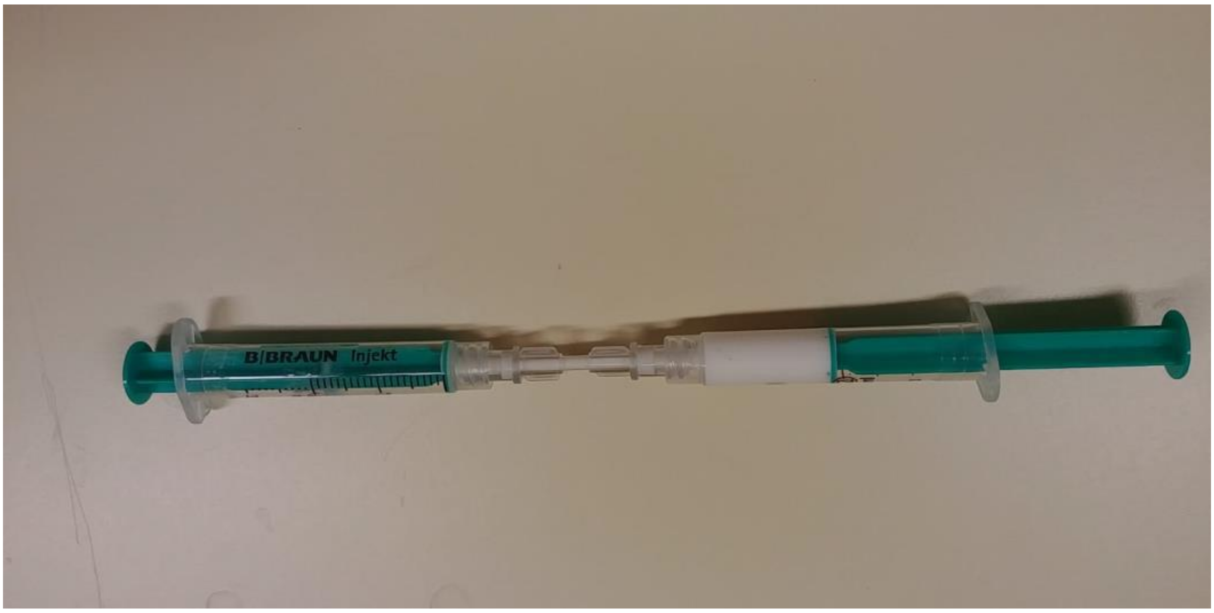

Supplementary Figure S2. The appearance of the emulsion that was formed by the two-syringe and i-connector method according to the instructions of the supplier (Seppic S.A., France).

Supplementary Table S1. The characteristics of the 20 different neoantigen peptides.

Peptide	Amino acid sequence	$[M+H]^+$ monoisotopic expected mass	pI	Cysteine content
FRM001P01	GCHLFSKRKCSQNKQSKRGKSRFYLSWRPL	3625.9	11.6	2
FRM001P02	SVTNTSLAHELWKVPLHLKTSLLRPAHLRD	3432.9	10.6	0
FRM001P03	RESSRPRGEETKRDQELEIAVGPEEAKLCL	3425.7	4.7	1
FRM001P04	SSDELRNGKMKAFLSLARFQILNTKELKTT	3439.9	10.6	0
FRM001P05	FMLIKNPMNVKNVERPLKCMATLPNIRKFI	3559.0	11.0	1
FRM001P06	LVRNPMCVSSVGKPSDMVQPLKPIREFIGA	3268.7	9.9	1
FRM001P09	REDNGLPMLSTNAMSWKTISAMEKRGARET	3380.6	9.9	0
FRM001P10	NTDPSCSCVTPLDFNHCLKNREDNGLPMLS	3320.5	4.4	3
FRM001P11	SAMEKRGARETQQQRKRVFRGRGVNSAK	3231.7	12.5	0
FRM001P12	CKECGRPLDVVHTLLHIRAFMLIKNPMNVK	3475.9	9.8	2
FRM001P13	GVDYASCGHLVKIWPRRMNRSDVCTVWS	3235.6	9.1	2
FRM001P14	RHSPNGNGPRSQHHSLITTNTDPSCSCVT	3103.4	8.3	2
FRM001P16	ERPQDTHSCLPVREEDPGLRVRWRLMIASV	3545.8	7.2	1
FRM001P17	AAEVRDPAVRLPLQGLPGQDLRGAHPAQA	3003.6	7.9	0
FRM001P18	GQDLRGAHPAQAHAGDALPGAQGGPPDLHR	2967.5	6.3	0
FRM001P19	HQLGQVRLRTATVLRPGAAAGRALPLVDAEL	3249.9	12.0	0
FRM001P20	AAGRALPLVDAELPADAAAEVRDPAVRLPL	3037.7	4.2	0
FRM001P21	DGWRLLPGVSRVQMESSRGRRRQGRQLRGQ	3521.9	12.7	0
FRM001P22	DLNVERALRVRGLPRLHQLGQVRLRTATVL	3450.0	12.5	0
FRM001P23	VTLEERLDKACEPGVDYDGQTGVIHFQPRG	3329.6	4.4	1

Supplementary Table S2. The long-term stability plan of the peptide mixtures that were produced under GMP conditions.

T = weeks [1]	t=0	t=2 w	t=4 w	t=8 w	t=12 w	t=16 w	t=32 w [2]
Peptide Identity	X	X	X	X	X	X	X
Peptide Purity	X	X	X	X	X	X	X
Peptide peak ratio	X	X	X	X	X	X	X
Bacterial Endotoxins	X	-	-	-	-	-	X
Sterility	X	-	-	-	-	-	X
Appearance	X	X	X	X	X	X	X
pH	X	-	-	X	X	X	X
Osmolality	X	-	-	X	X	X	X
In-use stability	X	X	X	X	X	X	X

[1] : The stability samples stored at -80 °C were analyzed on timepoint T=8 weeks and every timepoint after that.

[2] : The sterility and endotoxin tests were only performed on the stability samples stored at -25 °C due to the limited amount of samples. For these tests, 8 vials (of the 32-34 vials) were needed according to the Ph. Eur. 2.6.1.

Supplementary Table S3. The release, in-use, and end of shelf life specifications for the stability study of the peptide mixtures.

Test	Release specification	End of shelf life specification
Identity	$[M+H]^+$ monoisotopic expected mass ± 2 amu	$[M+H]^+$ monoisotopic expected mass ± 2 amu
Peptide peak ratio [1]	Report result	Report result
Peptide Purity [2]	≥ 85 % area	≥ 85 % area
Impurities (%) [3]	Report relative retention time (RRT) and % area of total	Report RRT and % area of total
Peptide Ratio	Report result	Report result
Bacterial Endotoxins	< 20 EU/mL	< 20 EU/mL
Sterility	Sterile	Sterile
Appearance	Clear colorless solution	Clear colorless solution
pH	6-8	6-8
Osmolality	270-310 mOsm/kg	270-310 mOsm/kg

[1] : Peptide peak ratio is reported as the % area of the individual peptide chromatogram peak in relation to the sum of all peak areas of the individual peptides (= 100%).
[2] : Peptide purity is the sum of the main peptide peak areas divided by the total observed area on the chromatogram of the test sample.
[3] : Any observed chromatogram peak of ≥ 0.40% not corresponding to the main peptide peak areas.

Supplementary Table S4. The results of the filter experiment. Unfiltered and filtered test samples of the peptide mixture batches FRM001-M1, FRM001-M2, FRM001-M3, and FRM001-M4 were analyzed to investigate potential filter effects.

**FRM001M1**				
**Test**		**Specification**	**Unfiltered**	**Filtered**
**Identity**	FRM001P01	3625.9 ± 2 u	3626.9 u	3626.9 u
	FRM001P04	3439.9 ± 2 u	3439.9 u	3439.9 u
	FRM001P05	3559.0 ± 2 u	3560.0 u	3560.0 u
	FRM001P14	3103.4 ± 2 u	3103.4 u	3103.4 u
	FRM001P19	3249.9 ± 2 u	3249.9 u	3249.9 u
	FRM001P21	3521.9 ± 2 u	3521.9 u	3521.9 u
**Peak ratio**	FRM001P01	Report result	14.8	15.0
	FRM001P04	Report result	16.3	16.1
	FRM001P05	Report result	25.2	25.2
	FRM001P14	Report result	10.9	10.8
	FRM001P19	Report result	22.8	23.1
	FRM001P21	Report result	9.9	9.8
**Peptide purity**		≥ 85 % area	98%	98%
**FRM001M2**				
**Identity**	FRM001P03	3425.7 ± 2 u	3425.8 u	3425.7 u
	FRM001P10	3320.5 ± 2 u	3320.5 u	3320.5 u
	FRM001P11	3231.7 ± 2 u	3231.8 u	3231.8 u
	FRM001P12	3475.9 ± 2 u	3475.9 u	3475.9 u
	FRM001P18	2967.5 ± 2 u	2967.5 u	2967.5 u
	FRM001P22	3450.0 ± 2 u	3450.1 u	3450.1 u
**Peak ratio**	FRM001P03	Report result	7.5	9.2
	FRM001P10	Report result	6.1	9.1
	FRM001P11	Report result	13.8	13.7
	FRM001P12	Report result	27.7	24.6
	FRM001P18	Report result	21.8	21.4
	FRM001P22	Report result	23.0	22.1
**Peptide purity**		≥ 85 % area	95%	95%
**FRM001M3**				
**Identity**	FRM001P02	3432.9 ± 2 u	3433.9 u	3434.0 u
	FRM001P05	3559.0 ± 2 u	3559.9 u	3559.9 u
	FRM001P06	3268.7 ± 2 u	3268.6 u	3268.7 u
	FRM001P09	3380.6 ± 2 u	3380.8 u	3380.6 u
	FRM001P18	2967.5 ± 2 u	2867.2 u	2967.2 u
	FRM001P23	3329.6 ± 2 u	3329.5 u	3329.6 u
**Peak ratio**	FRM001P02	Report result	18.6	18.7
	FRM001P05	Report result	22.2	22.7
	FRM001P06	Report result	16.9	16.4
	FRM001P09	Report result	14.2	14.2
	FRM001P18	Report result	16.3	16.2
	FRM001P23	Report result	11.9	11.8
**Peptide purity**		≥ 85 % area	96%	97%
**FRM001M4**				
**Identity**	FRM001P01	3625.9 ± 2 u	3625.8 u	3625.8 u
	FRM001P11	3231.7 ± 2 u	3231.8 u	3231.5 u
	FRM001P13	3235.6 ± 2 u	3235.5 u	3235.4 u
	FRM001P16	3545.8 ± 2 u	3546.0 u	3545.7 u
	FRM001P17	3003.6 ± 2 u	3003.4 u	3003.4 u
	FRM001P20	3037.7 ± 2 u	3037.4 u	3037.9 u
**Peak ratio**	FRM001P01	Report result	14.9	14.4
	FRM001P11	Report result	11.3	11.5
	FRM001P13	Report result	14.1	14.2
	FRM001P16	Report result	14.1	13.6
	FRM001P17	Report result	20.1	20.3
	FRM001P20	Report result	25.5	25.7
**Peptide purity**		≥ 85 % area	97%	97%

Supplementary Table S5. The chromatograms of the filtered and unfiltered test samples of the peptide mixtures FRM001-M1 and FRM001-M2.

Batch	Unfiltered	Filtered
FRM001-M1	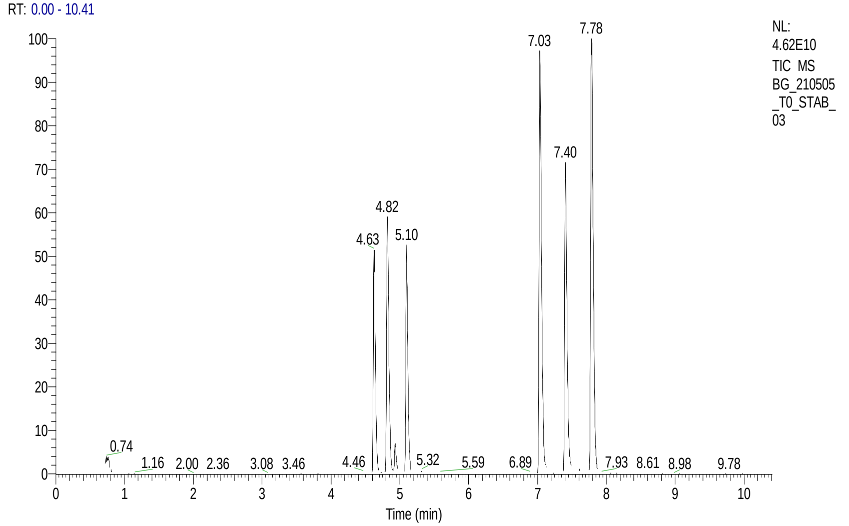	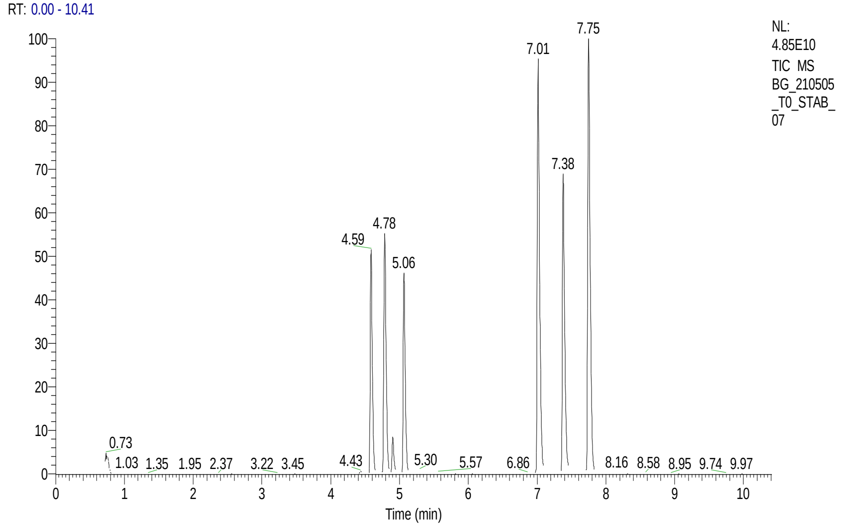
FRM001-M2	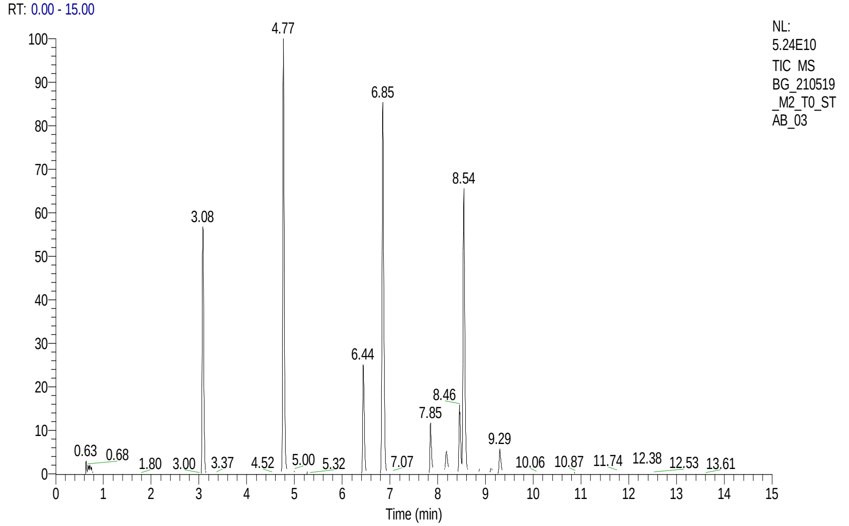	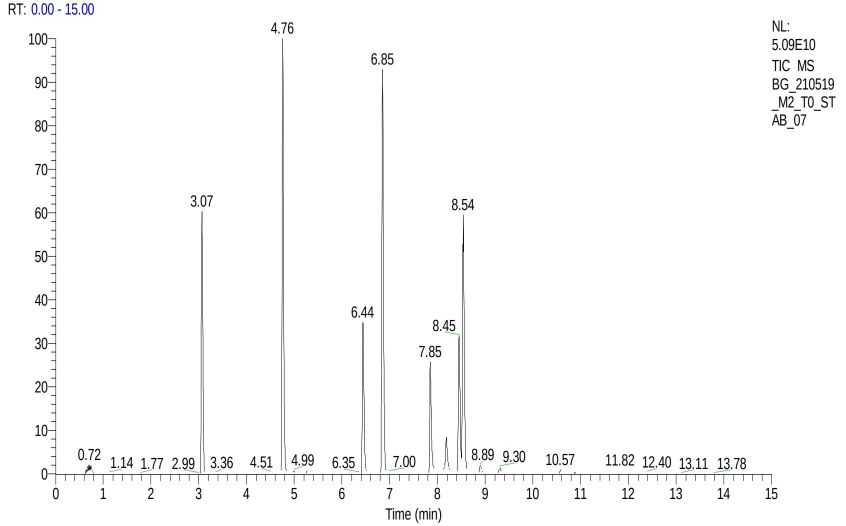

Supplementary Table S6. The chromatograms of the filtered and unfiltered test samples of the peptide mixtures FRM001-M3 and FRM001-M4.

Batch	Unfiltered	Filtered
FRM001-M3	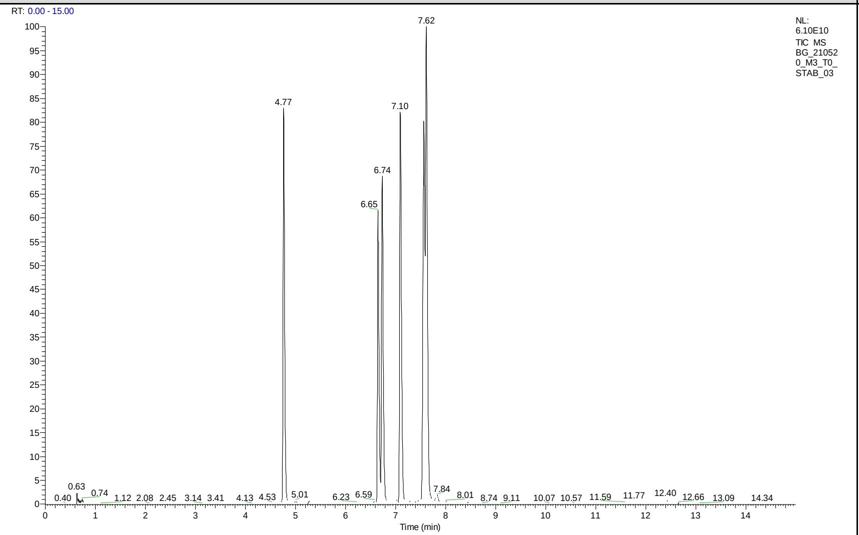	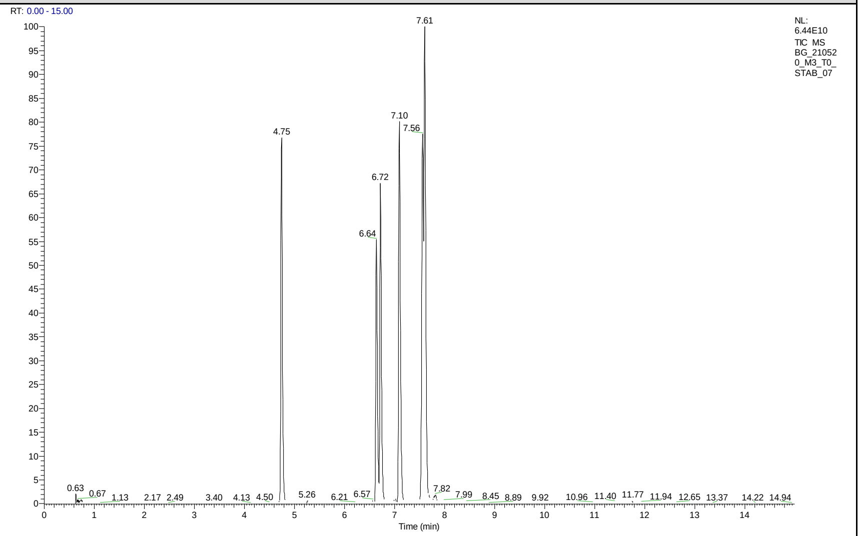
FRM001-M4	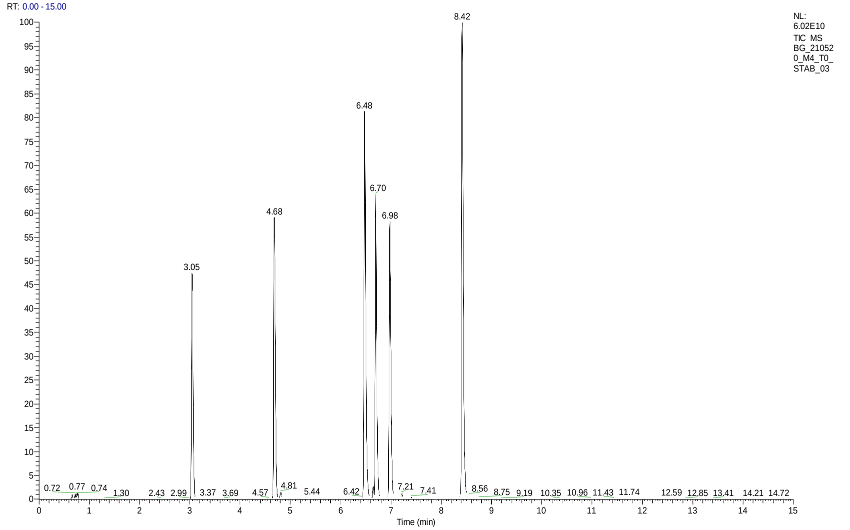	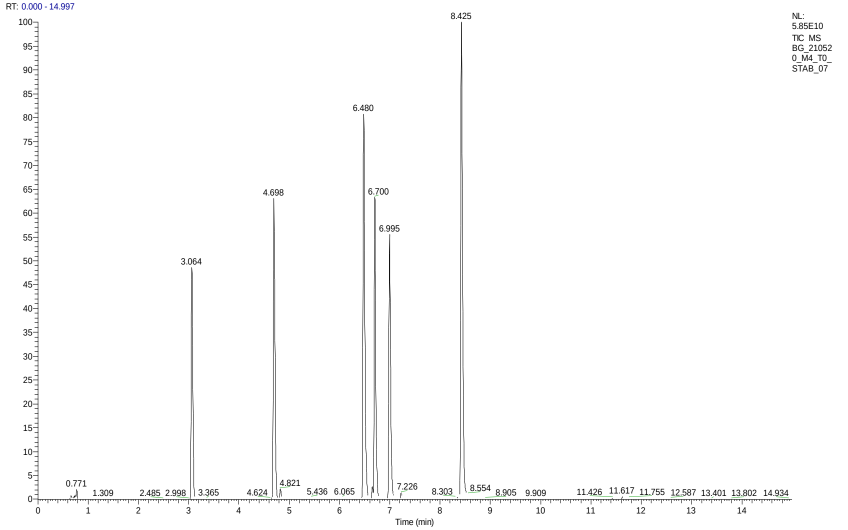

Supplementary Table S7 (part 1/2). The stability results of the FRM001-M2 peptide mixture stored at -25°C or -80°C.

**Test**		**Specification**	**t=0w**	**t=2w -25 °C**	**t=4w -25 °C**	**t=8w -25 °C**	**t=8w -80 °C**	**t=12w -25 °C**	**t=12w -80 °C**
Identity	FRM001P03	3425.7 ± 2 u	3425.7	3425.7	3425.7	3425.7	3425.7	3425.7	3425.7
	FRM001P10	3320.5 ± 2 u	3320.5	3320.5	3320.5	3320.5	3320.5	3320.5	3320.5
	FRM001P11	3231.7 ± 2 u	3231.8	2131.8	3231.8	3231.8	3231.8	3231.8	3231.8
	FRM001P12	3475.9 ± 2 u	3475.9	3475.8	3475.8	3475.9	3475.9	3475.9	3475.9
	FRM001P18	2967.5 ± 2 u	2967.5	2967.5	2967.5	2967.5	2967.5	2967.5	2967.5
	FRM001P22	3450.0 ± 2 u	3450.1	3450.1	3450.1	3450.1	3450.1	3450.1	3450.1
Peak ratio (%)	FRM001P03	Report result	9.2	9.2	10.5	9.9	10.3	11.3	11.0
	FRM001P10	Report result	9.1	7.2	11.5	11.0	12.9	14.2	13.8
	FRM001P11	Report result	13.7	14.7	14.1	14.9	14.8	13.5	14.2
	FRM001P12	Report result	24.6	22.0	19.5	19.6	20.5	18.1	20.0
	FRM001P18	Report result	21.4	22.1	21.5	21.5	19.8	20.7	19.9
	FRM001P22	Report result	22.1	24.2	22.9	23.1	21.3	22.2	21.2
Peptide purity		≥ 85% area	95%	95%	90%	89%	95%	95%	96%
Impurities (%) [1]		Report result	0.76 (RRT 0.24)<br>0.47 (RRT 2.91)	0.42 (RRT 0.23)<br>0.43 (RRT 2.91)<br>0.52 (RRT 3.46)	0.50 (RRT 2.43)<br>0.50 (RRT 3.44)<br>2.23 (RRT 3.83)<br>0.57 (RRT 3.90)<br>0.71 (RRT 3.96)<br>0.76 (RRT 4.05)<br>2.88 (RRT 4.07)	0.81 (RRT 0.21)<br>0.95 (RRT 0.22)<br>0.71 (RRT 0.24)<br>0.44 (RRT 0.64)<br>0.46 (RRT 2.48)<br>0.49 (RRT 3.44)<br>1.08 (RRT 3.54)<br>0.82 (RRT 3.61)<br>0.63 (RRT 3.78)<br>0.47 (RRT 3.85)<br>1.25 (RRT 4.07)	0.54 (RRT 0.23)<br>0.72 (RRT 3.45)	0.45 (RRT 0.57)<br>0.45 (RRT 0.61)<br>0.79 (RRT 2.44)<br>0.41 (RRT 3.41)<br>0.56 (RRT 4.03)	Not detected
Bacterial Endotoxins		< 20 EU/mL	< 0.5 EU/mL	n/a	n/a	n/a	n/a	n/a	n/a
Sterility		Sterile	Sterile	n/a	n/a	n/a	n/a	n/a	n/a
Appearance		Clear colorless solution	Clear colorless solution	Clear color-less solution	Clear colorless solution	Clear colorless solution	Clear colorless solution	Clear colorless solution	Clear colorless solution
pH		6-8	7	n/a	n/a	7	7	7	7
Osmolality (mOsm/kg)		270-310	296	n/a	n/a	300	300	296	298

[1]: Result is reported as retention time relative to FRM001P11 (first eluting peptide) and % area of the total sum of areas if the impurity peak area is ≥ 0.40%.

Supplementary Table S7 (continued part 2/2). The stability results of the FRM001-M2 peptide mixture stored at -25°C and -80°C.

**Test**		**Specification**	**t=0w**	**t=16w -25 °C**	**t=16w -80 °C**	**t=32w -25 °C**	**t=32w -80 °C**
Identity	FRM001P03	3425.7 ± 2 u	3425.7	3425.7	3425.7	3425.7	3425.7
	FRM001P10	3320.5 ± 2 u	3320.5	3320.5	3320.5	3320.5	3320.5
	FRM001P11	3231.7 ± 2 u	3231.8	3231.8	3231.8	3231.8	3231.8
	FRM001P12	3475.9 ± 2 u	3475.9	3475.9	3475.9	3475.9	3475.9
	FRM001P18	2967.5 ± 2 u	2967.5	2967.5	2967.5	2967.5	2967.5
	FRM001P22	3450.0 ± 2 u	3450.1	3450.1	3450.1	3450.1	3450.1
Peak ratio (%)	FRM001P03	Report result	9.2	10.2	10.3	10.2	10.1
	FRM001P10	Report result	9.1	9.6	10.4	10.8	10.7
	FRM001P11	Report result	13.7	13.2	13.4	12.8	13.1
	FRM001P12	Report result	24.6	17.9	19.5	20.3	22.6
	FRM001P18	Report result	21.4	25.5	24.1	22.3	21.8
	FRM001P22	Report result	22.1	23.6	22.3	23.6	21.8
Peptide purity		≥ 85% area	95%	86%	87%	90%	88%
Impurities (%) [1]		Report result	0.76 (RRT 0.24)<br>0.47 (RRT 2.91)	1.17 (RRT 0.82)<br>0.41 (RRT 0.35)<br>0.42 (RRT 2.45)<br>0.92 (RRT 3.66)<br>0.80 (RRT 3.83)<br>0.53 (RRT 3.87)<br>0.59 (RRT 3.90)<br>1.44 (RRT 4.06)	0.40 (RRT 0.36)<br>0.78 (RRT 0.82)<br>0.47 (RRT 3.64)<br>2.21 (RRT 3.70)<br>0.93 (RRT 3.76)<br>0.51 (RRT 3.81)<br>0.53 (RRT 3.81)<br>1.85 (RRT 3.87)<br>2.99 (RRT 4.00)<br>0.56 (RRT 4.08)	0.74 (RRT 0.89)<br>0.53 (RRT 3.02)<br>0.78 (RRT 4.40)<br>0.59 (RRT 4.55)<br>2.15 (RRT 4.97)<br>0.63 (RRT 5.20)<br>0.50 (RRT 5.34)	0.44 (RRT 4.36)<br>1.20 (RRT 4.41)<br>0.52 (RRT 4.59)<br>0.81 (RRT 4.94)<br>0.64 (RRT 4.99)<br>1.75 (RRT 5.20)<br>1.39 (RRT 5.23)<br>0.86 (RRT 5.27)<br>0.47 (RRT 5.32)
Bacterial Endotoxins		< 20 EU/mL	< 0.5 EU/mL	n/a	n/a	<0.5 EU/ml	n/a
Sterility		Sterile	Sterile	n/a	n/a	Sterile	n/a
Appearance		Clear colorless solution	Clear colorless solution	Clear colorless solution	Clear colorless solution	Clear colorless solution	Clear colorless solution
pH		6-8	7	7	7	7	7
Osmolality (mOsm/kg)		270-310	296	308	308	295	292

[1]: Result is reported as retention time relative to FRM001P11 (first eluting peptide) and % area of the total sum of areas if the impurity peak area is ≥ 0.40%.

Supplementary Table S8 (part 1/2). The stability results of the FRM001-M3 peptide mixture stored at -25°C and -80°C.

Test		Specification	t=0w	t=2w -25°C	t=4w -25°C	t=8w -25°C	t=8w -80°C	t=12w -25°C	t=12w -80°C
Identity	FRM001P02	3432.9 ± 2 u	3434.0	3432.9	3432.9	3432.9	3432.9	3432.9	3432.9
	FRM001P05	3559.0 ± 2 u	3559.9	3559.9	3559.9	3559.9	3559.9	3559.9	3559.9
	FRM001P06	3268.7 ± 2 u	3268.7	3269.7	3268.8	3269.7	3269.7	3269.7	3269.7
	FRM001P09	3380.6 ± 2 u	3380.6	3380.7	3380.7	3380.6	3380.6	3380.6	3380.6
	FRM001P18	2967.5 ± 2 u	2967.2	2967.5	2967.5	2967.1	2967.1	2967.1	2967.1
	FRM001P23	3329.6 ± 2 u	3329.6	3329.6	3329.6	3329.6	3329.6	3329.6	3329.6
Peak ratio (%)	FRM001P02	Report result	18.7	18.8	18.6	19.9	20.2	19.1	19.8
	FRM001P05	Report result	22.7	24.3	21.2	20.9	13.1	22.6	12.4
	FRM001P06	Report result	16.4	14.4	17.8	14.2	22.4	14.8	25.1
	FRM001P09	Report result	14.2	14.3	14.3	15.1	14.8	15.1	14.6
	FRM001P18	Report result	16.2	16.1	16.3	16.6	17.0	15.8	15.6
	FRM001P23	Report result	11.8	12.1	12.1	13.3	12.4	12.6	12.4
Peptide purity		≥ 85% area	97%	96%	95%	96%	94 %	97%	97%
Impurities (%)*		Report result	0.45 (RRT 1.65)	1.21 (RRT 1.65)	0.58 (RRT 0.16)<br>0.48 (RRT 2.40)<br>0.73 (RRT 2.48)<br>0.58 (RRT 2.62)	0.47 (RRT 0.13)<br>0.49 (RRT 0.15)<br>0.45 (RRT 1.64)	0.43 (RRT 0.13)<br>0.48 (RRT 2.43)<br>0.46 (RRT 2.58)<br>0.71 (RRT 2.62)	Not detected	Not detected
Bacterial Endotoxins		< 20 EU/mL	<0.05 EU/mL	n/a	n/a	n/a	n/a	n/a	n/a
Sterility		Sterile	Sterile	n/a	n/a	n/a	n/a	n/a	n/a
Appearance		Clear colorless solution	Clear colorless solution	Clear colorless solution	Clear colorless solution	Clear colorless solution	Clear colorless solution	Clear colorless solution	Clear colorless solution
pH**		6-8	7	n/a	n/a	7	7	7	7
Osmolality (mOsm/kg) **		270-310	293	n/a	n/a	297	298	297	297

* Result is reported as retention time relative to FRM001P18 (first eluting peptide) and % area of the total sum of areas if the impurity peak area is ≥ 0.40%.

** These tests will only be performed on the engineering batches of the Drug Product.

Supplementary Table S8 (continued part 2/2). The stability results of the FRM001-M3 peptide mixture stored at -25°C and -80°C.

**Test**		**Specification**	**t=0w**	**t=16w -25°C**	**t=16w -80°C**	**t=32w -25°C**	**t=32w -80°C**
Identity	FRM001P02	3432.9 ± 2 u	3434.0	3432.9	3432.9	3432.9	3432.9
	FRM001P05	3559.0 ± 2 u	3559.9	3559.9	3559.9	3559.9	3559.9
	FRM001P06	3268.7 ± 2 u	3268.7	3269.7	3269.7	3269.7	3269.7
	FRM001P09	3380.6 ± 2 u	3380.6	3380.6	3380.6	3380.6	3380.6
	FRM001P18	2967.5 ± 2 u	2967.2	2967.1	2967.1	2967.1	2967.1
	FRM001P23	3329.6 ± 2 u	3329.6	3329.6	3329.6	3329.6	3329.6
Peak ratio (%)	FRM001P02	Report result	18.7	18.5	18.3	19.3	19.3
	FRM001P05	Report result	22.7	20.4	23.4	22.3	22.7
	FRM001P06	Report result	16.4	21.6	18.8	16.3	16.0
	FRM001P09	Report result	14.2	12.7	12.6	14.1	14.1
	FRM001P18	Report result	16.2	16.4	16.5	17.0	16.7
	FRM001P23	Report result	11.8	10.4	10.3	11.0	11.1
Peptide purity		≥ 85% area	97%	92%	92%	93%	94%
Impurities (%) [1]		Report result	0.45 (RRT 1.65)	0.52 (RRT 0.53)	0.43 (RRT 0.09)<br>0.41 (RRT 0.23)<br>0.53 (RRT 0.53)<br>0.86 (RRT 2.58)	0.51 (RRT 2.56)<br>1.35 (RRT 2.80)<br>0.97 (RRT 2.90)<br>1.15 (RRT 2.91)<br>0.57 (RRT 2.92)	0.55 (RRT 2.81)<br>1.01 (RRT 2.89)<br>1.37 (RRT 2.91)
Bacterial Endotoxins		< 20 EU/mL	<0.05 EU/mL	n/a	n/a	<0.5 EU/ml	n/a
Sterility		Sterile	Sterile	n/a	n/a	Sterile	n/a
Appearance		Clear colorless solution	Clear colorless solution	Clear colorless solution	Clear colorless solution	Clear colorless solution	Clear colorless solution
pH		6-8	7	7	7	7	7
Osmolality (mOsm/kg)		270-310	293	299	297	278	289

[1]: Result is reported as retention time relative to FRM001P18 (first eluting peptide) and % area of the total sum of areas if the impurity peak area is ≥ 0.40%.

Supplementary Table S9 (part 1/2). The stability results of the FRM001-M4 peptide mixture stored at -25°C and -80°C.

Test		Specification	t=0w	t=2w -25 °C	t=4w -25 °C	t=8w -25 °C	t=8w -80 °C	t=12w -25 °C	t=12w -80 °C
Identity	FRM001P01	3625.9 ± 2 u	3625.8	3625.8	3625.9	3625.7	3625.7	3625.7	3625.7
	FRM001P11	3231.7 ± 2 u	3231.5	3231.8	3232.8	3232.8	3232.8	3232.8	3232.8
	FRM001P13	3235.6 ± 2 u	3235.4	3236.4	3236.6	3235.6	3235.6	3235.6	3235.6
	FRM001P16	3545.8 ± 2 u	3545.7	3545.7	3545.8	3545.8	3545.8	3545.8	3545.8
	FRM001P17	3003.6 ± 2 u	3003.4	3003.6	3003.6	3003.6	3003.6	3003.6	3003.6
	FRM001P20	3037.7 ± 2 u	3037.9	3037.6	3037.7	3037.7	3037.7	3037.7	3037.7
Peak ratio (%)	FRM001P01	Report result	14.4	14.5	15.4	15.1	15.2	15.1	14.8
	FRM001P11	Report result	11.5	11.7	12.1	12.7	12.9	12.8	12.3
	FRM001P13	Report result	14.2	13.6	14.6	14.7	14.9	14.4	14.6
	FRM001P16	Report result	13.6	13.1	13.4	13.1	13.4	13.6	13.4
	FRM001P17	Report result	20.3	21.0	20.4	20.4	19.9	19.5	20.0
	FRM001P20	Report result	25.7	26.0	24.0	24.0	23.7	24.6	24.8
Peptide purity		≥ 85% area	97%	96%	93%	91%	96%	96 %	98%
Impurities (%) [1]		Report result	0.43 (RRT 0.23)	0.46 (RRT 0.23)<br>0.56 (RRT 3.84)	1.13 (RRT 3.63)<br>1.10 (RRT 3.85)<br>0.60 (RRT 4.08)	0.59 (RRT 0.21)<br>0.85 (RRT 0.23)<br>0.44 (RRT 3.56)<br>0.52 (RRT 3.63)<br>0.55 (RRT 3.74)<br>0.92 (RRT 3.85)<br>2.81 (RRT 4.03)	0.72 (RRT 0.25)<br>0.40 (RRT 2.21)	0.41 (RRT 3.73)<br>0.57 (RRT 4.08)	Not detected
Bacterial Endotoxins		< 20 EU/mL	<0.05 EU/mL	n/a	n/a	n/a	n/a	n/a	n/a
Sterility		Sterile	Sterile	n/a	n/a	n/a	n/a	n/a	n/a
Appearance		Clear colorless solution	Clear colorless solution	Clear colorless solution	Clear colorless solution	Clear colorless solution	Clear colorless solution	Clear colorless solution	Clear colorless solution
pH		6-8	7	n/a	n/a	7	7	7	7
Osmolality (mOsm/kg)		270-310	294	n/a	n/a	297	298	298	298

[1]: Result is reported as retention time relative to FRM001P11 (first eluting peptide) and % area of the total sum of areas if the impurity peak area is ≥ 0.40%.

Supplementary Table S9 (continued part 2/2). The stability results of the FRM001-M4 peptide mixture stored at -25°C and -80°C.

Test		Specification	t=0w	t=16w -25 °C	t=16w -80 °C	t=32w -25 °C	t=32w -80 °C
Identity	FRM001P01	3625.9 ± 2 u	3625.8	3625.7	3625.7	3625.7	3625.7
	FRM001P11	3231.7 ± 2 u	3231.5	3232.8	3232.8	3232.8	3232.8
	FRM001P13	3235.6 ± 2 u	3235.4	3235.6	3235.6	3235.6	3235.6
	FRM001P16	3545.8 ± 2 u	3545.7	3545.8	3545.8	3545.8	3545.8
	FRM001P17	3003.6 ± 2 u	3003.4	3003.6	3003.6	3003.6	3003.6
	FRM001P20	3037.7 ± 2 u	3037.9	3037.7	3037.7	3037.7	3037.7
Peak ratio (%)	FRM001P01	Report result	14.4	12.5	12.5	12.9	14.7
	FRM001P11	Report result	11.5	11.0	11.1	10.8	12.1
	FRM001P13	Report result	14.2	13.3	13.1	13.6	13.9
	FRM001P16	Report result	13.6	10.6	10.8	11.0	13.1
	FRM001P17	Report result	20.3	21.9	22.0	21.8	20.9
	FRM001P20	Report result	25.7	30.7	30.5	29.9	25.3
Peptide purity		≥ 85% area	97%	91%	91%	90%	88%
Impurities (%) [1]		Report result	0.43 (RRT 0.23)	0.53 (RRT 0.26)<br>0.72 (RRT 0.82)	0.49 (RRT 0.36)<br>0.69 (RRT 0.82)	0.42 (RRT 4.50)<br>0.47 (RRT 4.75)<br>1.73 (RRT 4.97)<br>1.62 (RRT 5.17)<br>0.42 (RRT 5.14)<br>0.82 (RRT 5.20)	0.72 (RRT 4.52)<br>0.60 (RRT 4.59)<br>0.73 (RRT 4.61)<br>0.44 (RRT 4.65)<br>0.64 (RRT 4.66)<br>0.42 (RTT 4.74)<br>0.73 (RRT 4.79)<br>0.50 (RRT 4.85)<br>0.97 (RRT 4.91)<br>1.10 (RRT 5.19)<br>1.56 (RRT 5.23)<br>0.95 (RRT 5.27)
Bacterial Endotoxins		< 20 EU/mL	<0.05 EU/mL	n/a	n/a	<0.5 EU/ml	n/a
Sterility		Sterile	Sterile	n/a	n/a	Sterile	n/a
Appearance		Clear colorless solution	Clear colorless solution	Clear colorless solution	Clear colorless solution	Clear colorless solution	Clear colorless solution
pH		6-8	7	7	7	7	7
Osmolality (mOsm/kg)		270-310	294	298	304	299	282

[1]: Result is reported as retention time relative to FRM001P11 (first eluting peptide) and % area of the total sum of areas if the impurity peak area is ≥ 0.40%.

Supplementary Table S10 (part 1/2). The results of the in-use stability of the FRM001-M1 batch. After the t=0 h analysis, the same sample was stored at room temperature and analyzed again after 2 h and 4 h. For readability, only the 0 h and 4h data are presented; all 2h data were similar and complied.

Test		t=0w	t=2w -25 °C		t=4w -25 °C		t=8w -25 °C		t=8w -80 °C		t=12w -25 °C		t=12w -80 °C	
		**0h**	**0h**	**4h**	**0h**	**4h**	**0h**	**4h**	**0h**	**4h**	**0h**	**4h**	**0h**	**4h**
Peak ratio %	FRM001P01	15.0	15.0	15.2	14.6	14.8	15.2	15.3	15.0	15.2	15.9	15.3	15.5	15.3
	FRM001P04	16.1	15.7	15.8	16.7	16.7	16.7	16.6	16.6	16.4	16.4	16.9	17.3	16.9
	FRM001P05	25.2	25.1	24.6	25.4	25.1	25.3	25.7	25.3	25.6	24.5	24.6	26.8	24.6
	FRM001P14	10.8	11.2	11.3	10.5	10.5	10.9	10.8	10.8	10.7	11.9	11.2	7.6	11.2
	FRM001P19	23.1	22.8	22.8	22.1	22.1	21.3	20.9	21.7	21.5	19.9	21.2	21.6	21.2
	FRM001P21	9.8	10.2	10.3	10.7	10.7	10.6	10.7	10.7	10.6	11.3	10.9	11.1	10.9
Peptide purity		98%	98%	98%	95%	96%	94%	96%	96%	97%	94%	96%	98%	96%
Impurities[1]		0.47 (RRT 0.16)	0.41 (RRT1.70)	0.53 (RTT 0.17)	0.62 (RRT 2.60)<br>0.67 (RRT 2.71)<br>0.51 (RRT 2.79)	0.62 (RRT 0.14)<br>0.49 (RRT 0.15)	0.46 (RRT 0.15)<br>0.65 (RRT 2.39)<br>0.41 (RRT 2.43)<br>0.63 (RRT 2.60)<br>0.60 (RRT 2.63)<br>0.58 (RRT 2.70)<br>0.48 (RRT 2.76)	0.48 (RRT 0.17)	0.53 (RRT 0.16)<br>0.48 (RRT 2.58)<br>0.51 (RRT 2.72)	0.41 (RRT 0.16)	0.51 (RRT 2.51)<br>0.69 (RRT 2.56)<br>0.46 (RRT 2.62<br>1.60 (RRT 2.73)	0.43 (RRT 0.14)<br>0.48 (RRT 0.16)	0.44 (RRT 0.16)	0.41 (RRT 0.16)

[1]: Result is reported as retention time relative to FRM001P14 (first eluting peptide) and % area of the total sum of areas if the impurity peak area is ≥ 0.40%.

Supplementary Table S10 (continued part 2/2). The results of the in-use stability of the FRM001-M1 batch. After the t=0 h analysis, the same sample was stored at room temperature and analyzed again after 2 h and 4 h. For readability, only the 0 h and 4h data are presented; all 2h data were similar and complied.

Test		t=0w	t=16w -25 °C		t=16w -80 °C		t=32w -25 °C		t=32w -80 °C	
		0h	0h	4h	0h	4h	0h	4h	0h	4h
Peak ratio %	FRM001P01	15.0	15.4	15.5	15.3	15.6	3626.9	14.1	14.2	14.1
	FRM001P04	16.1	17.2	17.0	16.6	16.6	3439.9	17.1	16.7	16.7
	FRM001P05	25.2	25.6	25.5	25.7	25.3	3560.0	25.2	24.9	25.2
	FRM001P14	10.8	10.1	10.5	10.7	10.6	3103.4	10.9	11.8	11.5
	FRM001P19	23.1	21.5	21.2	21.4	21.2	3249.9	21.8	21.4	21.6
	FRM001P21	9.8	10.1	10.3	10.3	10.6	3521.9	10.8	11.1	11.0
Peptide purity		98%	97%	98%	97%	98%	92%	94%	95%	95%
Impurities[1]		0.47 (RRT 0.16)	0.40 (RRT 2.58)<br>0.61 (RRT 2.73)	Not detected	0.52 (RRT 2.63)	Not detected	0.81 (RTT 2.74)<br>0.55 (RRT 2.79)<br>0.88 (RRT 2.84)<br>1.12 (RRT 2.98)<br>2.09 (RRT 3.01)<br>1.11 (RRT 3.12)<br>0.92 (RRT 3.16)<br>0.53 (RRT 3.21)	0.40 (RRT 2.91)<br>1.37 (RRT 3.12)<br>0.96 (RRT 3.14)<br>0.56 (RRT 3.16)<br>0.70 (RRT 3.21)	0.44 (RRT 2.96)<br>0.78 (RRT 3.12)<br>0.65 (RRT 3.14)<br>0.56 (RRT 3.16)	0.48 (RRT 3.00)<br>0.91 (RRT 3.13)<br>0.66 (RRT 3.16)<br>0.56 (RRT 3.17)

[1]: Result is reported as retention time relative to FRM001P14 (first eluting peptide) and % area of the total sum of areas if the impurity peak area is ≥ 0.40%.

Supplementary Table S11 (part 1/2). The results of the in-use stability of the FRM001-M2 batch. After the t=0 h analysis, the same sample was stored at room temperature and analyzed again after 2 h and 4 h. For readability, only the 0 h and 4h data are presented; all 2h data were similar and complied.

**Test**		**t=0w**	**t=2w -25 °C**		**t=4w -25 °C**		**t=8w -25 °C**		**t=8w -80 °C**		**t=12w -25 °C**		**t=12w -80 °C**	
		**0h**	**0h**	**4h**	**0h**	**4h**	**0h**	**4h**	**0h**	**4h**	**0h**	**4h**	**0h**	**4h**
Peak ratio %	FRM001P03	9.2	9.2	9.7	10.5	10.4	9.9	10.3	10.3	10.5	11.3	11.1	11.0	11.0
	FRM001P10	9.1	7.2	8.9	11.5	12.1	11.0	12.1	12.9	12.0	14.2	13.7	13.8	13.2
	FRM001P11	13.7	14.7	13.8	14.1	13.5	14.9	14.2	14.8	14.7	13.5	13.2	14.2	14.1
	FRM001P12	24.6	22.0	23.0	19.5	20.5	19.5	21.0	20.5	21.0	18.1	19.7	20.0	20.6
	FRM001P18	21.4	22.1	21.5	21.5	21.5	21.5	20.4	19.8	20.1	20.7	20.3	19.9	19.9
	FRM001P22	22.1	24.2	23.1	22.9	21.9	23.1	22.1	21.6	21.8	22.2	21.9	21.2	21.2
Peptide purity		95%	95%	94%	90%	94%	89%	94%	95%	95%	95%	95%	96%	96%
Impurities[1]		0.76<br>(RRT 0.24)<br>0.47<br>(RRT 2.91)	0.42<br>(RRT 0.23)<br>0.43<br>(RRT 2.91)<br>0.52<br>(RRT 3.46)	0.56<br>(RRT 0.23)	0.50<br>(RRT 2.43)<br>0.50<br>(RRT 3.44)<br>2.23<br>(RRT 3.83)<br>0.57<br>(RRT 3.90)<br>0.71<br>(RRT 3.96)<br>0.76<br>(RRT 4.05)<br>2.88<br>(RRT 4.07)	0.59<br>(RRT 0.58)<br>0.45<br>(RRT 2.44)<br>0.55<br>(RRT 3.45)<br>1.16<br>(RRT 3.89)	0.81<br>(RRT 0.21)<br>0.95<br>(RRT 0.22)<br>0.71<br>(RRT 0.24)<br>0.44<br>(RRT 0.64)<br>0.46<br>(RRT 2.48)<br>0.49<br>(RRT 3.44)<br>1.08<br>(RRT 3.54)<br>0.82<br>(RRT 3.61)<br>0.63<br>(RRT 3.79)<br>0.47<br>(RRT 3.85)<br>1.25<br>(RRT 0.64)	0.47<br>(RRT 0.24)<br>0.42<br>(RRT 0.60)<br>0.45<br>(RRT 2.48)<br>0.48<br>(RRT 3.45)	0.54<br>(RRT 0.23)<br>0.72<br>(RRT 3.45)	0.47<br>(RRT 0.21)<br>0.63<br>(RRT 0.24)<br>0.59<br>(RRT 3.45)	0.45<br>(RRT 0.57)<br>0.45<br>(RRT 0.61)<br>0.79<br>(RRT 2.44)<br>0.41<br>(RRT 3.41)<br>0.56<br>(RRT 4.03)	0.41<br>(RRT 0.59)<br>0.46<br>(RRT0.63)<br>0.49<br>(RRT 0.67)<br>0.66<br>(RRT 2.46)<br>0.40<br>(RRT 2.55)	Not detected	Not detected

[1]: Result is reported as retention time relative to FRM001P11 (first eluting peptide) and % area of the total sum of areas if the impurity peak area is ≥ 0.40%.

Supplementary Table S11 (continued part 2/2). The results of the in-use stability of the FRM001-M2 batch. After the t=0 h analysis, the same sample was stored at room temperature and analyzed again after 2 h and 4 h. For readability, only the 0 h and 4h data are presented; all 2h data were similar and complied.

**Test**		**t=0w**	**t=16w -25 °C**		**t=16w -80 °C**		**t=32w -25 °C**		**t=32w -80 °C**	
		**0h**	**0h**	**4h**	**0h**	**4h**	**0h**	**4h**	**0h**	**4h**
Peak ratio %	FRM001P03	9.2	10.2	10.5	10.3	10.3	10.2	10.1	10.1	9.7
	FRM001P10	9.1	9.6	10.2	10.4	10.1	10.8	9.6	10.7	9.3
	FRM001P11	13.7	13.2	12.7	13.4	13.4	12.8	12.8	13.1	12.9
	FRM001P12	24.6	17.9	18.9	19.5	20.0	20.3	22.7	22.6	24.2
	FRM001P18	21.4	25.5	24.7	24.1	24.0	22.3	22.3	21.8	21.9
	FRM001P22	22.1	23.6	23.0	22.3	22.2	23.6	22.5	21.8	21.9
Peptide purity		95%	86%	90%	87%	89%	90%	90%	88%	92%
Impurities[1]		0.76 (RRT 0.24)<br>0.47 (RRT 2.91)	1.17 (RRT 0.82)<br>0.41 (RRT 0.35)<br>0.42 (RRT 2.45)<br>0.92 (RRT 3.66)<br>0.80 (RRT 3.83)<br>0.53 (RRT 3.87)<br>0.59 (RRT 3.90)<br>1.44 (RRT 4.06)	0.48 (RRT0.30)<br>1.01 (RRT 0.82)<br>0.41 (RRT 2.46)	0.40 (RRT 0.36)<br>0.78 (RRT 0.82)<br>0.47 (RRT 3.64)<br>2.21 (RRT 3.70)<br>0.93 (RRT 3.76)<br>0.51 (RRT 3.81)<br>1.85 (RRT 3.87)<br>0.53 (RRT 3.81)<br>2.99 (RRT 4.00)<br>0.56 (RRT 4.08)	0.43 (RRT 0.23)<br>0.82 (RRT 0.82)<br>1.40 (RRT 4.08)	0.74 (RRT 0.89)<br>0.53 (RRT 3.02)<br>0.78 (RRT 4.40)<br>0.59 (RRT 4.55)<br>2.15 (RRT 4.97)<br>0.63 (RRT 5.20)<br>0.50 (RRT 5.34)	0.57 (RRT 0.89)<br>1.00 (RRT 5.20)<br>1.26 (RRT 5.23)<br>0.68 (RRT 5.28)<br>0.41 (RRT 3.16)<br>0.70 (RRT 4.94)<br>0.41 (RRT 5.31)	0.44 (RRT4.36)<br>1.20 (RRT4.41)<br>0.52 (RRT4.59)<br>0.81 (RRT4.94)<br>0.64 (RRT4.99)<br>1.75 (RRT5.20)<br>1.39 (RRT5.23)<br>0.86 (RRT5.27)<br>0.47 (RRT5.32)	0.42 (RRT 4.41)<br>0.75 (RRT 5.20)<br>1.42 (RRT 5.23)<br>0.80 (RRT 5.26)<br>0.72 (RRT 4.96)

[1]: Result is reported as retention time relative to peptide FRM001P11 (first eluting peptide) and % area of the total sum of areas if the impurity peak area is ≥ 0.40%.

Supplementary Table S12 (part 1/2). The results of the in-use stability of the FRM001-M3 batch. After the t=0 h analysis, the same sample was stored at room temperature and analyzed again after 2 h and 4 h. For readability, only the 0 h and 4h data are presented; all 2h data were similar and complied.

**Test**		**t=0w**	**t=2w -25 °C**		**t=4w -25 °C**		**t=8w -25 °C**		**t=8w -80 °C**		**t=12w -25 °C**		**t=12w -80 °C**	
		**0h**	**0h**	**4h**	**0h**	**4h**	**0h**	**4h**	**0h**	**4h**	**0h**	**4h**	**0h**	**4h**
Peak ratio %	FRM001P02	18.7	18.8	19.0	18.6	18.6	19.9	19.8	20.2	20.6	19.1	19.3	19.8	19.6
	FRM001P05	22.7	24.3	21.9	21.2	19.5	14.2	12.3	13.1	12.9	14.8	13.8	12.4	17.6
	FRM001P06	16.4	14.4	16.5	17.8	18.9	20.9	22.8	22.4	21.2	22.6	23.9	25.1	19.9
	FRM001P09	14.2	14.3	14.3	14.3	14.3	15.1	15.2	14.8	15.5	15.1	15.1	14.6	14.7
	FRM001P18	16.2	16.1	16.2	16.3	16.4	16.6	16.8	17.0	17.3	15.8	15.5	15.6	15.7
	FRM001P23	11.8	12.1	12.1	12.1	12.2	13.3	13.1	12.4	12.5	12.6	12.3	12.4	12.5
Peptide purity		97%	96%	96%	95%	97%	89%	94%	94%	96%	97%	97%	97%	97%
Impurities[1]		0.45<br>(RRT<br>1.65)	1.21<br>(RRT<br>1.65)	0.61<br>(RRT 1.64)	0.58<br>(RRT 0.16)<br>0.48<br>(RRT 2.40)<br>0.73<br>(RRT 2.48)<br>0.58<br>(RRT 2.62)	0.48<br>(RRT 1.65)	0.46<br>(RRT<br>0.13)<br>0.52<br>(RRT<br>0.15)	0.47<br>(RRT 0.13)<br>0.49<br>(RRT 0.15)<br>0.45<br>(RRT 1.64)	0.43<br>(RRT 0.13)<br>0.48<br>(RRT 2.42)<br>0.46<br>(RRT 2.58)<br>0.71<br>(RRT 2.62)	0.60<br>(RRT0.16)<br>0.50<br>(RRT1.62)	Not detected	Not detected	Not detected	Not detected

[1]: Result is reported as retention time relative to peptide FRM001P18 (first eluting peptide) and % area of the total sum of areas if the impurity peak area is ≥ 0.40%.

Supplementary Table S12 (continued part 2/2). The results of the in-use stability of the FRM001-M3 batch. After the t=0 h analysis, the same sample was stored at room temperature and analyzed again after 2 h and 4 h. For readability, only the 0 h and 4h data are presented; all 2h data were similar and complied.

**Test**		**t=0w**	**t=16w -25 °C**		**t=16w -80 °C**		**t=32w -25 °C**		**t=32w -80 °C**	
		**0h**	**0h**	**4h**	**0h**	**4h**	**0h**	**4h**	**0h**	**4h**
Peak ratio %	FRM001P02	18.7	18.5	18.4	18.3	18.5	19.3	19.4	19.3	19.7
	FRM001P05	22.7	20.4	24.1	23.4	23.7	22.3	21.8	22.7	22.0
	FRM001P06	16.4	21.6	18.3	18.8	18.5	16.3	16.4	16.0	16.4
	FRM001P09	14.2	12.7	12.6	12.6	12.6	14.1	13.9	14.1	13.9
	FRM001P18	16.2	16.4	16.5	16.5	16.5	17.0	16.9	16.7	16.8
	FRM001P23	11.8	10.4	10.2	10.3	10.1	11.0	11.6	11.1	11.1
Peptide purity		97%	92%	97%	92%	93%	93%	92%	94%	94%
Impurities[1]		0.45<br>(RRT 1.65)	0.52 (RRT<br>0.53)	0.54 (RRT 0.53)	0.43 (RRT 0.09)<br>0.41 (RRT 0.23)<br>0.53 (RRT 0.53)<br>0.86 (RRT 2.58)	0.52 (RRT 0.53)	0.51<br>(RRT2.56)<br>1.35<br>(RRT2.80)<br>0.97<br>(RRT2.90)<br>1.15<br>(RRT2.91)<br>0.57<br>(RRT2.92)	0.46 (RRT 2.57)<br>1.11 (RRT 2.83)<br>2.87 (RRT 2.91)<br>0.57 (RRT 2.93)<br>0.87 (RRT 2.79)<br>0.95 (RRT 2.88)	0.55 (RRT 2.81)<br>1.01 (RRT 2.89)<br>1.37 (RRT 2.91)	0.83<br>(RRT2.92)<br>1.18<br>(RRT2.94)<br>0.68<br>(RRT2.96)

[1]: Result is reported as retention time relative to peptide FRM001P18 (first eluting peptide) and % area of the total sum of areas if the impurity peak area is ≥ 0.40%.

Supplementary Table S13 (part 1/2). The results of the in-use stability of the FRM001-M4 batch. After the t=0 h analysis, the same sample was stored at room temperature and analyzed again after 2 h and 4 h. For readability, only the 0 h and 4h data are presented; all 2h data were similar and complied.

**Test**		**t=0w**	**t=2w -25 °C**		**t=4w -25 °C**		**t=8w -25 °C**		**t=8w -80 °C**		**t=12w -25 °C**		**t=12w -80 °C**	
		**0h**	**0h**	**4h**	**0h**	**4h**	**0h**	**4h**	**0h**	**4h**	**0h**	**4h**	**0h**	**4h**
Peak ratio %	FRM001P01	14.4	14.5	14.7	15.4	15.5	15.1	15.3	15.2	15.5	15.1	14.8	14.8	15.1
	FRM001P11	11.5	11.7	11.7	12.1	12.0	12.7	12.9	12.9	12.9	12.8	12.3	12.3	12.1
	FRM001P13	14.2	13.6	13.4	14.6	14.5	14.7	14.4	14.9	14.7	14.4	14.6	14.6	14.4
	FRM001P16	13.6	13.1	13.0	13.4	13.2	13.1	12.9	13.4	13.4	13.6	13.4	13.4	13.5
	FRM001P17	20.3	21.0	21.2	20.4	20.6	20.4	20.3	19.9	20.2	19.5	20.0	20.0	20.1
	FRM001P20	25.7	26.0	26.0	24.0	24.3	24.0	24.2	23.7	23.4	24.6	24.8	24.8	24.8
Peptide purity		97%	96%	96%	93%	97%	91%	96%	96%	96%	96%	97%	98%	98%
Impurities[1]		0.43 (RRT 0.23)	0.46 (RRT 0.23)<br>0.56 (RRT 3.84)	0.48 (RRT3.84)	1.13 (RRT3.63)<br>1.10 (RRT3.85)<br>0.60 (RRT4.08)	0.54 (RRT 0.25)	0.60 (RRT 0.21)<br>0.85 (RRT 0.23)<br>0.44 (RRT 3.56)<br>0.52 (RRT 3.62)<br>0.55 (RRT 3.74)<br>0.92 (RRT 3.85)<br>2.81 (RRT 4.03)	0.67 (RRT 0.23)<br>0.45 (RRT 2.21)	0.72 (RRT 0.25)<br>0.40 (RRT 2.21)	0.48 (RRT 0.23)<br>0.71 (RRT 0.25)<br>0.43 (RRT 2.20)	0.41 (RRT 3.73)<br>0.57 (RRT 4.08)	Not detected	Not detected	Not detected

[1]: Result is reported as retention time relative to peptide FRM001P11 (first eluting peptide) and % area of the total sum of areas if the impurity peak area is ≥ 0.40%.

Supplementary Table S13 (continued part 2/2). The results of the in-use stability of the FRM001-M4 batch. After the t=0 h analysis, the same sample was stored at room temperature and analyzed again after 2 h and 4 h. For readability, only the 0 h and 4h data are presented; all 2h data were similar and complied.

**Test**		**t=0w**	**t=16w -25 °C**		**t=16w -80 °C**		**t=32w -25 °C**		**t=32w -80 °C**	
		**0h**	**0h**	**4h**	**0h**	**4h**	**0h**	**4h**	**0h**	**4h**
Peak ratio %	FRM001P01	14.4	12.5	12.4	12.5	12.4	12.9	13.1	14.7	14.9
	FRM001P11	11.5	11.0	11.2	11.1	11.1	10.8	11.1	12.1	12.1
	FRM001P13	14.2	13.3	13.1	13.1	13.0	13.6	13.5	13.9	13.4
	FRM001P16	13.6	10.6	10.5	10.8	10.8	11.0	11.2	13.1	12.8
	FRM001P17	20.3	21.9	22.0	22.0	22.1	21.8	22.1	21.0	21.1
	FRM001P20	25.7	30.7	30.7	30.5	30.5	29.9	28.9	25.3	25.7
Peptide purity		97%	91%	91%	91%	91%	90%	92%	88%	93%
Impurities[1]		0.43<br>(RRT 0.23)	0.53 (RRT 0.26)<br>0.72 (RRT 0.82)	0.72<br>(RRT0.82)<br>0.52<br>(RRT0.36)	0.49<br>(RRT0.36)<br>0.69<br>(RRT0.82)	0.40 (RRT 0.36)<br>0.71 (RRT 0.82)	1.73 (RRT4.97)<br>1.62 (RRT5.17)<br>0.82 (RRT5.20)<br>0.47 (RRT4.75)<br>0.42 (RRT5.14)<br>0.42 (RRT4.50)	0.40 (RRT 4.49)<br>0.98 (RRT 4.98)<br>1.46 (RRT 5.16)<br>0.78 (RRT 5.18)<br>0.73 (RRT 5.23)<br>0.41 (RRT 4.56)<br>0.44 (RRT 5.27)	0.72 (RRT4.52)<br>0.60 (RRT4.59)<br>0.73 (RRT4.61)<br>0.44 (RRT4.65)<br>0.64 (RRT4.66)<br>0.42 (RTT4.74)<br>0.73 (RRT4.79)<br>0.50 (RRT4.85)<br>0.97 (RRT4.91)<br>1.10 (RRT5.19)<br>1.56 (RRT5.23)<br>0.95 (RRT5.27)	0.89 (RRT 5.20)<br>1.21 (RRT 5.24)<br>0.83 (RRT 5.28)<br>0.42 (RRT 4.48)

[1]: Result is reported as retention time relative to peptide FRM001P11 (first eluting peptide) and % area of the total sum of areas if the impurity peak area is ≥ 0.40%.
